# Hypermigration of macrophages through the concerted action of GRA effectors on NF-κB/p38 signaling and host chromatin accessibility potentiates *Toxoplasma* dissemination

**DOI:** 10.1128/mbio.02140-24

**Published:** 2024-08-29

**Authors:** Arne L. ten Hoeve, Matias E. Rodriguez, Martin Säflund, Valentine Michel, Lucas Magimel, Albert Ripoll, Tianxiong Yu, Mohamed-Ali Hakimi, Jeroen P. J. Saeij, Deniz M. Ozata, Antonio Barragan

**Affiliations:** 1 Department of Molecular Biosciences, The Wenner-Gren Institute, Stockholm University, Stockholm, Sweden; 2 Program in Bioinformatics and Integrative Biology, University of Massachusetts Medical School, Worcester, Massachusetts, USA; 3 Institute for Advanced Biosciences, INSERM U1209, CNRS UMR5309, Université Grenoble Alpes, Grenoble, France; 4 Department of Pathology, Microbiology, and Immunology, University of California Davis, Davis, California, USA; University of Geneva, Geneva, Switzerland

**Keywords:** mononuclear phagocyte, intracellular parasitism, host-pathogen, cell signaling pathway, immune cell migration

## Abstract

Mononuclear phagocytes facilitate the dissemination of the obligate intracellular parasite *Toxoplasma gondii*. Here, we report how a set of secreted parasite effector proteins from dense granule organelles (GRA) orchestrates dendritic cell-like chemotactic and pro-inflammatory activation of parasitized macrophages. These effects enabled efficient dissemination of the type II *T. gondii* lineage, a highly prevalent genotype in humans. We identify novel functions for effectors GRA15 and GRA24 in promoting CCR7-mediated macrophage chemotaxis by acting on NF-κB and p38 mitogen-activated protein kinase signaling pathways, respectively, with contributions by GRA16/18 and counter-regulation by effector TEEGR. Furthermore, GRA28 boosted chromatin accessibility and GRA15/24/NF-κB-dependent transcription at the *Ccr7* gene locus in primary macrophages. *In vivo*, adoptively transferred macrophages infected with wild-type *T. gondii* outcompeted macrophages infected with a GRA15/24 double mutant in migrating to secondary organs in mice. The data show that *T. gondii*, rather than being passively shuttled, actively promotes its dissemination by inducing a finely regulated pro-migratory state in parasitized human and murine phagocytes via co-operating polymorphic GRA effectors.

**IMPORTANCE:**

Intracellular pathogens can hijack the cellular functions of infected host cells to their advantage, for example, for intracellular survival and dissemination. However, how microbes orchestrate the hijacking of complex cellular processes, such as host cell migration, remains poorly understood. As such, the common parasite *Toxoplasma gondii* actively invades the immune cells of humans and other vertebrates and modifies their migratory properties. Here, we show that the concerted action of a number of secreted effector proteins from the parasite, principally GRA15 and GRA24, acts on host cell signaling pathways to activate chemotaxis. Furthermore, the protein effector GRA28 selectively acted on chromatin accessibility in the host cell nucleus to selectively boost host gene expression. The joint activities of GRA effectors culminated in pro-migratory signaling within the infected phagocyte. We provide a molecular framework delineating how *T. gondii* can orchestrate a complex biological phenotype, such as the migratory activation of phagocytes to boost dissemination.

## INTRODUCTION

Macrophages originate from embryonic progenitors or monocytes and are crucial cells for the innate immune response (1). Being typically sessile cells residing in peripheral tissues, macrophages maintain tissue homeostasis and combat infections with versatile responses, including phagocytosis (2). Conversely, many pathogens have developed strategies to survive and thrive within macrophages and also manipulate the diverse functions of phagocytes to their advantage (3, 4). Macrophages and dendritic cells (DCs) can be distinguished by transcriptional signatures, for example, ZBTB46, IRF4, and BATF3 expression, which also reflects ontology and tissue localization (5).

*Toxoplasma gondii* is an obligate intracellular pathogen commonly carried by humans and many other warm-blooded vertebrates (6). Following oral primary infection, *T. gondii* disseminates broadly in the organism to reach peripheral organs, including the central nervous system. While chronic carriage is chiefly asymptomatic, acute or reactivated infection can cause life-threatening disease in the developing fetus and in immunocompromised individuals (7, 8).

Colonization of the host is mediated by the tachyzoite stage of *T. gondii*. Being obligate intracellular, tachyzoites actively invade host cells (9) in peripheral tissues, including macrophages. The parasite makes use of infected phagocytes for systemic dissemination by a *Trojan horse* mechanism, in a parasite genotype-related fashion (10–12). Mononuclear phagocytes, including principally macrophages, DCs, monocytes, and microglia, are induced to migrate via activation of GABAergic signaling and MAP kinase activation (13–16). This migratory activation, termed hypermigratory phenotype (17), implicates secreted parasite effectors (18–21) and impacts the motility and chemotaxis of infected phagocytes (22–24).

The invasion of host cells by tachyzoites comprises a discharge of secretory organelles (25, 26). Within parasitized cells, the MYR1 secretory machinery ensures the transport of many dense granule proteins (GRAs) across the parasitophorous vacuole (PV), whereafter GRAs can traffic to the host cell cytosol and nucleus (27). GRA proteins present little or no homology to each other and are polymorphic among the clonal lineages of *T. gondii* (types I, II, and III) that predominate in Europe and North America. Type II strains are most prevalent in humans and animals used for meat consumption (28, 29).

GRA proteins bear important functions in the immunomodulatory impact of *T. gondii* on the host, such as activation of the NF-κB pathway and MAP kinase signaling in macrophages (30, 31) and chromatin remodeling, which impact transcription in the host cell (32, 33). Notably, recent findings identified a role for the chromatin remodeler-interacting effector GRA28 (type I) on the migratory activation of phagocytes. Among the features of this activation are the expression of chemokine receptor CCR7, the onset of chemotaxis, and systemic migration by parasitized macrophages in mice (21).

Here, we investigated the molecular machinery that imparts a DC-like migratory activation on parasitized macrophages. We show that the concerted action of polymorphic effectors regulates the pro-migratory activation of parasitized macrophages. Specifically, a set of secreted GRA proteins impacts NF-κB/p38 mitogen-activated protein kinase (MAPK) signaling and host chromatin accessibility to co-operatively promote CCR7-driven chemotaxis in infected macrophages and, thereby, potentiate parasite dissemination.

## RESULTS

### The effector GRA15 mediates a DC-like migratory activation in *T. gondii*-infected macrophages

**Fig 1:**
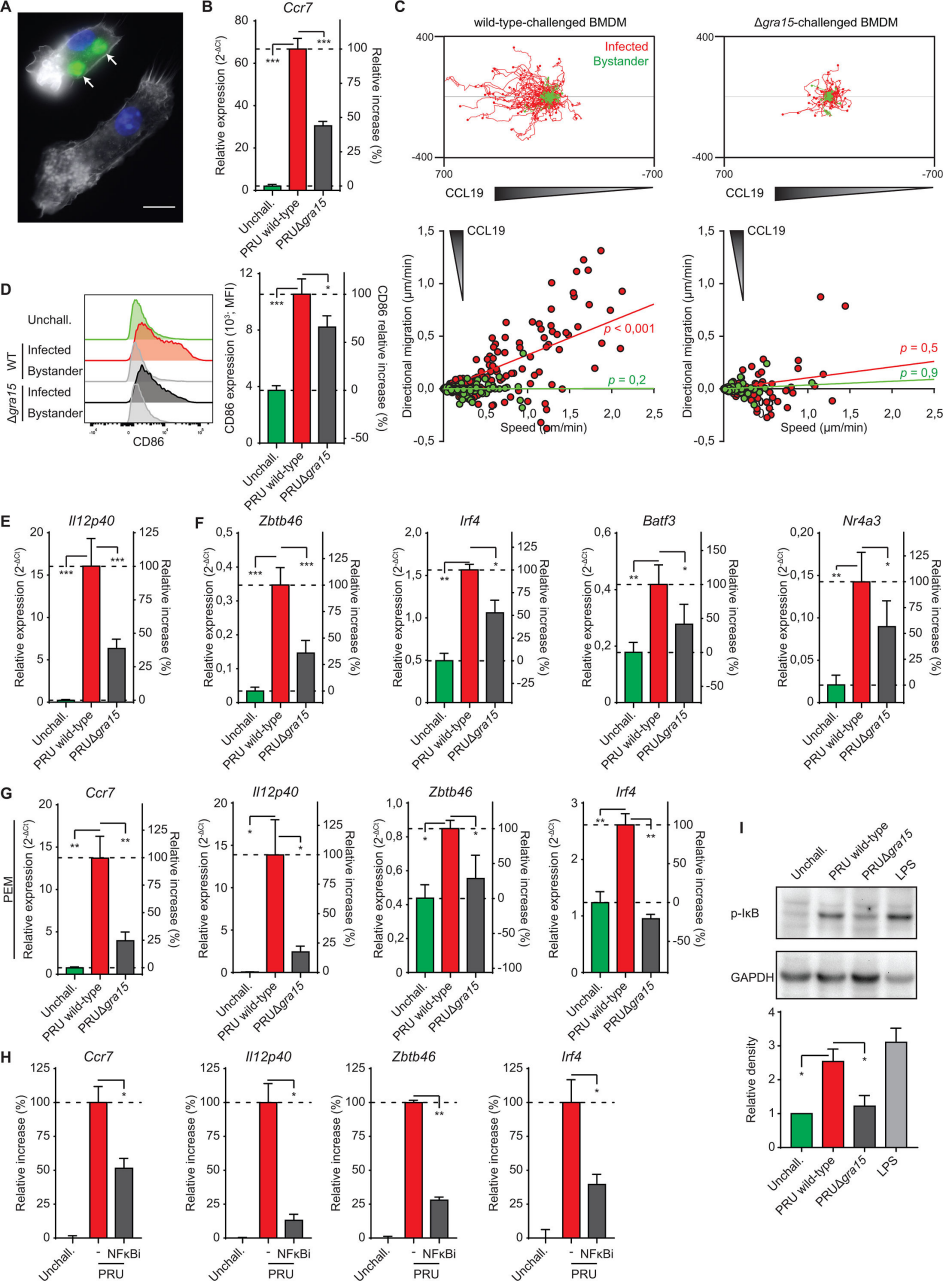
Phenotypes *T. gondii*-infected macrophages upon GRA15-deficiency and NF-κB inhibition. (**A**) The representative micrograph shows primary BMDMs stained for F-actin (phalloidin Alexa Fluor 594, white) and nuclei (DAPI, blue). Arrows indicate two intracellular vacuoles with replicating GFP-expressing type II *T. gondii* tachyzoites (green) 18 h post-challenge. Lower bystander cell is uninfected. Scale bar = 10 µm. (**B**) Quantitative PCR (qPCR) analysis of *Ccr7* cDNA from BMDMs challenged for 18 h with freshly egressed *T. gondii* type II wild-type and GRA15-deficient (Δ*gra15*) tachyzoites [PRU; Multiplicity of infection (MOI) 2]. For reference, macrophages were incubated in complete medium, unchallenged (unchall.). Displayed are relative expression (2^−ΔCt^) and the increase in expression relative to wild type (100%) and unchallenged (0%) conditions (mean + SEM; *n* = 4 independent experiments). (**C**) Motility plots depict the displacement of BMDMs challenged with freshly egressed *T. gondii* type II wild-type and GRA15-deficient (Δ*gra15*) tachyzoites (PRU; MOI 1) over 14 h in a collagen matrix with a CCL19 gradient as detailed in Materials and methods (scale indicates µm; *n* = 3). For each condition, directional migration (µm/min) toward the CCL19 source and speed (µm/min) of individual cells is displayed in graphs, with linear regression lines. Infected cells (GFP^+^, red) and non-infected bystander cells (GFP^−^, green) were analyzed. For each condition, *P*-values indicate the directional migration compared to hypothetical zero directionality (one-sample permutation test). (**D**) Flow cytometric analysis of anti-CD86 staining on BMDMs challenged for 18 h with freshly egressed GFP-expressing *T. gondii* type II wild-type (WT) and GRA15-deficient (Δ*gra15*) tachyzoites (PRU; MOI 1) or left unchallenged. Infected (GFP^+^) and bystander cells (GFP^−^) were analyzed. The bar graph displays the Mean fluorescence intensity (MFI) and the increase in expression relative to wild-type (100%) and unchallenged (0%) conditions (mean + SEM; *n* = 5). (**E** and **F**) qPCR analyses of *Il12p40* (**E**) or *Zbtb46*, *Irf4*, and *Nr4a3* (**F**) cDNA from BMDMs challenged and displayed as in (**B**), *n* = 4. (**G**) qPCR analyses of *Ccr7*, *Il12p40*, *Zbtb46*, and *Irf4* cDNA from resident peritoneal macrophages (PEMs) challenged and displayed as in (**B**), *n* = 3. (**H**) qPCR analyses of *Ccr7*, *Il12p40*, *Zbtb46*, and *Irf4* cDNA from BMDMs challenged for 18 h with GFP-expressing *T. gondii* type II wild-type tachyzoites with or without JSH-23 treatment (NFκBi). Displayed is the increase in expression relative to untreated unchallenged (0%) and wild-type (100%) challenged conditions (mean + SEM; *n* = 3). (**I**) Western blot analysis of phospho-IκBα ser32/36 (p-IκB) levels in BMDMs challenged for 5 h with freshly egressed *T. gondii* type II wild-type or GRA15-deficient (Δ*gra15*) tachyzoites (PRU, MOI 3) or LPS (10 ng/mL) or left unchallenged (unchall.). The bar graph displays the relative density of specific p-IκB signal relative to specific GAPDH signal (mean + SEM; *n* = 3). Statistical comparisons were made with ANOVA and Dunnett’s post-hoc (**B and D–I**) or one-sample permutation tests (B; *P ≤ 0.05, **P ≤ 0.01, ***P ≤ 0.001, and ns *P* > 0.05).

The infection of macrophages by *T. gondii* tachyzoites (type I) initiates a migratory activation, which encompasses the expression of DC-associated transcription factors, phenotypical changes, and the onset of CCR7-dependent chemotaxis (21). Because NF-κB positively regulates CCR7 expression in DCs (34) and the *T. gondii* protein GRA15 (type II) activates NF-κB (30), we hypothesized that GRA15 contributes to the migratory activation of parasitized macrophages. Bone marrow-derived macrophages (BMDMs) challenged with type II *T. gondii* tachyzoites (wild-type PRU; Fig. 1A) dramatically upregulated the expression of *Ccr7* mRNA (Fig. 1B). Interestingly, BMDMs challenged with GRA15-deficient tachyzoites (PRUΔ*gra15*) consistently exhibited a significantly decreased *Ccr7* expression (Fig. 1B), compared with wild-type-challenged BMDMs at similar infection frequencies (Fig. S1A). To functionally assess the putative impact of the induced *Ccr7*, we performed chemotaxis assays with the CCR7-ligand chemokine CCL19. Notably, wild-type PRU-infected BMDMs, but not bystander BMDMs, displayed a distinct migratory response toward the CCL19 source (Fig. 1C). In sharp contrast, chemotaxis to CCL19 was dramatically reduced in BMDMs challenged with PRUΔ*gra15* (Fig. 1C), while random hypermotility (22) with elevated velocity was maintained (Fig. S1B). Collectively, the findings identify a role for GRA15 in mediating CCR7-dependent chemotaxis, without measurable effects on bystander cells or on hypermotility (13).

Next, we assessed the impact of GRA15 on the expression of co-stimulatory molecules (CD40/80/86), MHCII, and *Il12p40* because they are also regulated by NF-κB (35, 36). *T. gondii* wild-type (PRU)-infected BMDMs, but not bystander BMDMs, robustly upregulated CD86 expression (Fig. 1D), while CD40, CD80, and MHCII were minorly affected (Fig. S1D and E). Notably, PRUΔ*gra15*-infected BMDMs expressed reduced CD80 and CD86 levels compared with wild-type-infected BMDMs. Furthermore, wild-type-challenged BMDMs upregulated *Il12p40* expression, which was significantly reduced in PRU*Δgra15*-challenged BMDMs (Fig. 1E). Finally, we determined the contribution of GRA15 to the expression of DC-associated transcription factors, normally not expressed or expressed at low level in macrophages (21). Consistently, BMDMs challenged with wild-type tachyzoites elevated the expression of *Zbtb46*, *Irf4*, *Batf3*, and *Nr4a3* and GRA15-deficiency significantly reduced the relative expression of these genes (Fig. 1F). Data were confirmed using primary peritoneal macrophages (PEM; Fig. 1G). In contrast, early growth response 1 (*Egr1*) expression, known to be GRA24 dependent (31, 37), was significantly enhanced by GRA15-deficiency (Fig. S1F). Furthermore, the reconstitution of GRA15 in mutants (PRUΔgra15 + GRA15) resulted in elevated *Ccr7* and *Il12p40* expression (Fig. S1G), confirming the role of GRA15. Next, cells were treated with separate NF-κB inhibitors targeting nuclear translocation of NF-κB or activator IκB kinase (IKK) (38, 39). Similar to both inhibitors, treated *T. gondii*-challenged BMDMs expressed significantly lower levels of *Ccr7*, *Il12p40*, *Zbtb46*, and *Irf4* than their untreated counterparts (Fig. 1H; Fig. S1H), thus mimicking the effects of GRA15-deficiency. Finally, we confirmed a GRA15-dependent S32/36 phosphorylation of the NF-κB cytoplasmic anchor IκB in *T. gondii*-challenged BMDMs (Fig. 1I). We conclude that in *T. gondii* type II (PRU)-infected macrophages, GRA15 contributes to the inductions of CCR7-dependent chemotaxis, *Il12p40*, and DC-associated transcription factors in an NF-κB-dependent manner.

### Impact of the MYR1-dependent effector GRA24 on the migratory activation of macrophages

**Fig 2:**
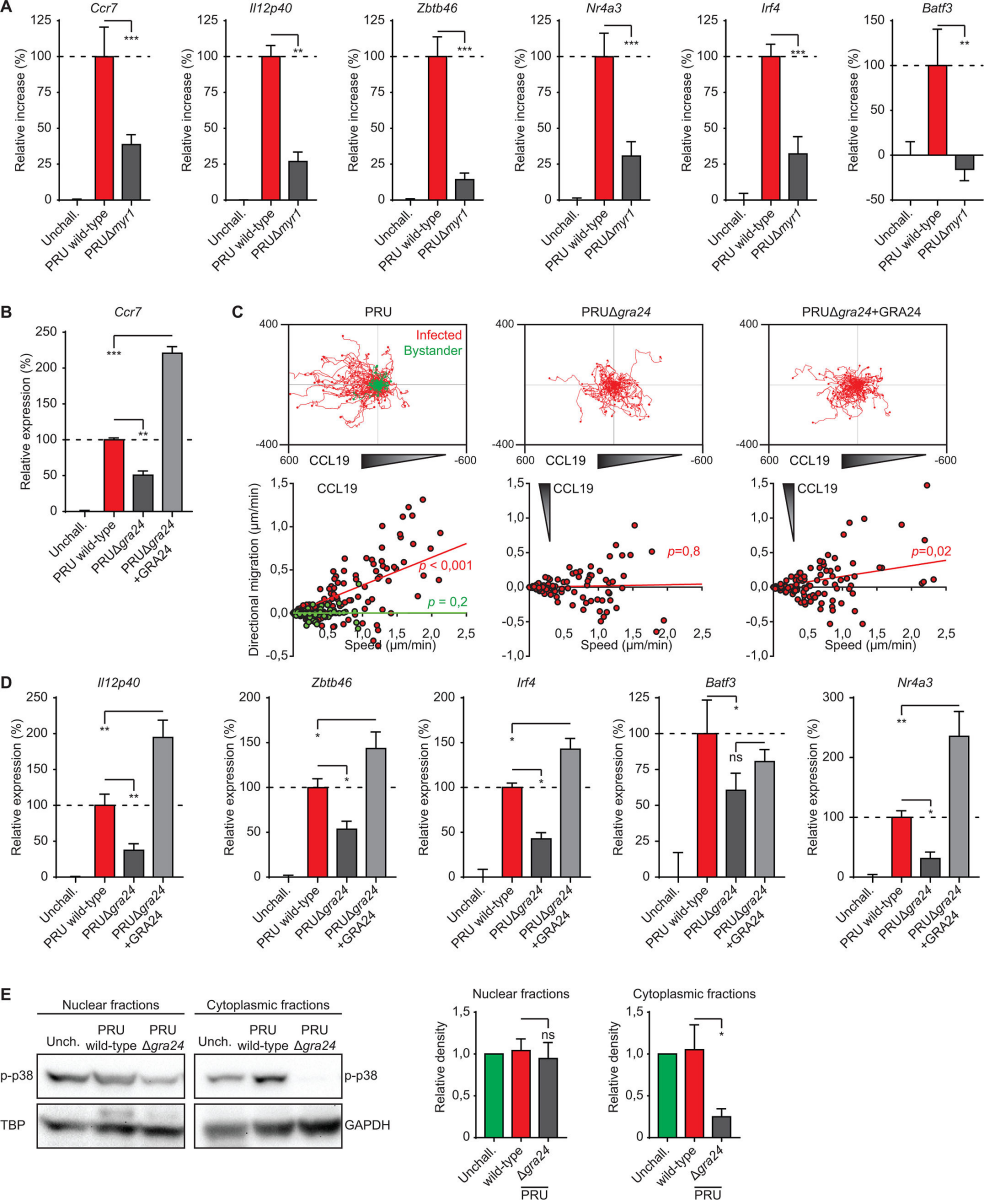
Phenotypes of *T. gondii*-infected macrophages upon MYR1 and GRA24 deficiency. (**A**) Quantitative PCR (qPCR) analyses of *Ccr7*, *Il12p40*, *Zbtb46*, *Nr4a3*, *Irf4*, and *Batf3* cDNA from BMDMs challenged for 18 h with freshly egressed *T. gondii* type II wild-type and MYR1-deficient (Δ*myr1*) tachyzoites (PRU; MOI 2) or left unchallenged (unchall.). Bar graphs display the increase in expression relative to untreated unchallenged (0%) and wild-type (100%) challenged conditions (mean + SEM; *n* = 4). (**B**) qPCR analysis of *Ccr7* cDNA from BMDMs challenged for 18 h with *T. gondii* type II wild-type, GRA24-deficient (Δ*gra24*), or GRA24-reconstituted (Δ*gra24* + GRA24) tachyzoites (PRU; MOI 2) or left unchallenged (unchall.), displayed as in (**A**), *n* = 3. (**C**) Motility plots depict the displacement of BMDMs challenged with freshly egressed *T. gondii* type II wild-type and GRA24-deficient (Δ*gra24*) tachyzoites (PRU; MOI 1) over 14 h in a collagen matrix with a CCL19 gradient as detailed in Materials and methods (scale indicates µm; *n* = 3). For each condition, directional migration (µm/min) toward the CCL19 source and speed (µm/min) of individual cells is displayed in graphs, with linear regression lines. Infected cells (GFP^+^, red) and non-infected bystander cells (GFP^−^, green) were analyzed. For each condition, *P*-values indicate the directional migration compared to hypothetical zero directionality (one-sample permutation test). (**D**) qPCR analyses of *Il12p40*, *Zbtb46*, *Irf4*, *Batf3*, and *Nr4a3* of BMDMs challenged and displayed as in (**B**). (**E**) Western blot analysis of p-p38 (Thr180/Tyr182) expression in cytoplasm- and nucleus-enriched fractions of BMDMs challenged for 5 h with wild-type and GRA24-deficient (Δ*gra24*) *T. gondii* type II tachyzoites (PRU, MOI 3). Bar graphs display the relative density of p-p38 signal relative to TATA-binding protein (TBP) or GAPDH signals (mean + SEM; *n* = 5). Statistical comparisons were made with ANOVA and Dunnett’s post-hoc tests (*P ≤ 0.05, **P ≤ 0.01, ***P ≤ 0.001, and ns *P* > 0.05).

Several *T. gondii*-derived proteins that modulate host cell signaling rely on the MYR1 secretory pathway for translocation to the host cell cytosol and nucleus (40). We recently identified a role for the MYR1-translocated effector GRA28 in the induction of DC-like migratory activation of macrophages by the type I RH strain (21). However, the fact that the type I RH strain lacks a functional GRA15 (30), and that the reduction of macrophage activation upon GRA15 deficiency (type II) was partial (Fig. 1), motivated the investigation of additional putative effectors. First, we confirmed that the inductions of *Ccr7*, *Il12p40*, *Zbtb46*, *Nr4a3*, *Irf4*, and *Batf3* were significantly reduced in macrophages challenged with type II MYR1-deficient *T. gondii* (PRUΔ*myr1*; Fig. 2A). Because p38 MAPK can act as a positive regulator of CCR7 expression in DCs (41), we hypothesized a contribution of the MYR1-secreted p38-activating effector GRA24 (27, 31) to the induction of *Ccr7-* and CCR7-dependent chemotaxis in infected macrophages. In line with this idea, GRA24-deficient (PRUΔ*gra24*) tachyzoites induced significantly lower levels of *Ccr7* (~50% reduction) compared with wild-type *T. gondii* and reconstitution (PRUΔgra24 + GRA24) resulted in elevated *Ccr7* expression (Fig. 2B). Consistent with the transcriptional data, PRUΔ*gra24*-infected BMDMs did not display chemotaxis toward CCL19, while chemotaxis was recovered in GRA24-reconstituted PRUΔ*gra24 T. gondii* (Fig. 2C). Similar to *Ccr7*, the expressions of *Il12p40*, *Zbtb46*, *Irf4*, *Batf3*, and *Nr4a3* were reduced upon challenge with PRUΔ*gra24* (Fig. 2D). Finally, we confirmed that, in *T. gondii*-challenged BMDMs, cytoplasmic phosphorylated p38 (p-p38) MAPK levels were maintained in a GRA24-dependent manner (Fig. 2E). Together, the data implicate GRA24/p38 in the migratory activation of macrophages and motivated a further investigation of MAPK signaling.

### Ribosomal S6 kinase regulates the migratory activation of *T. gondii*-infected macrophages

**Fig 3:**
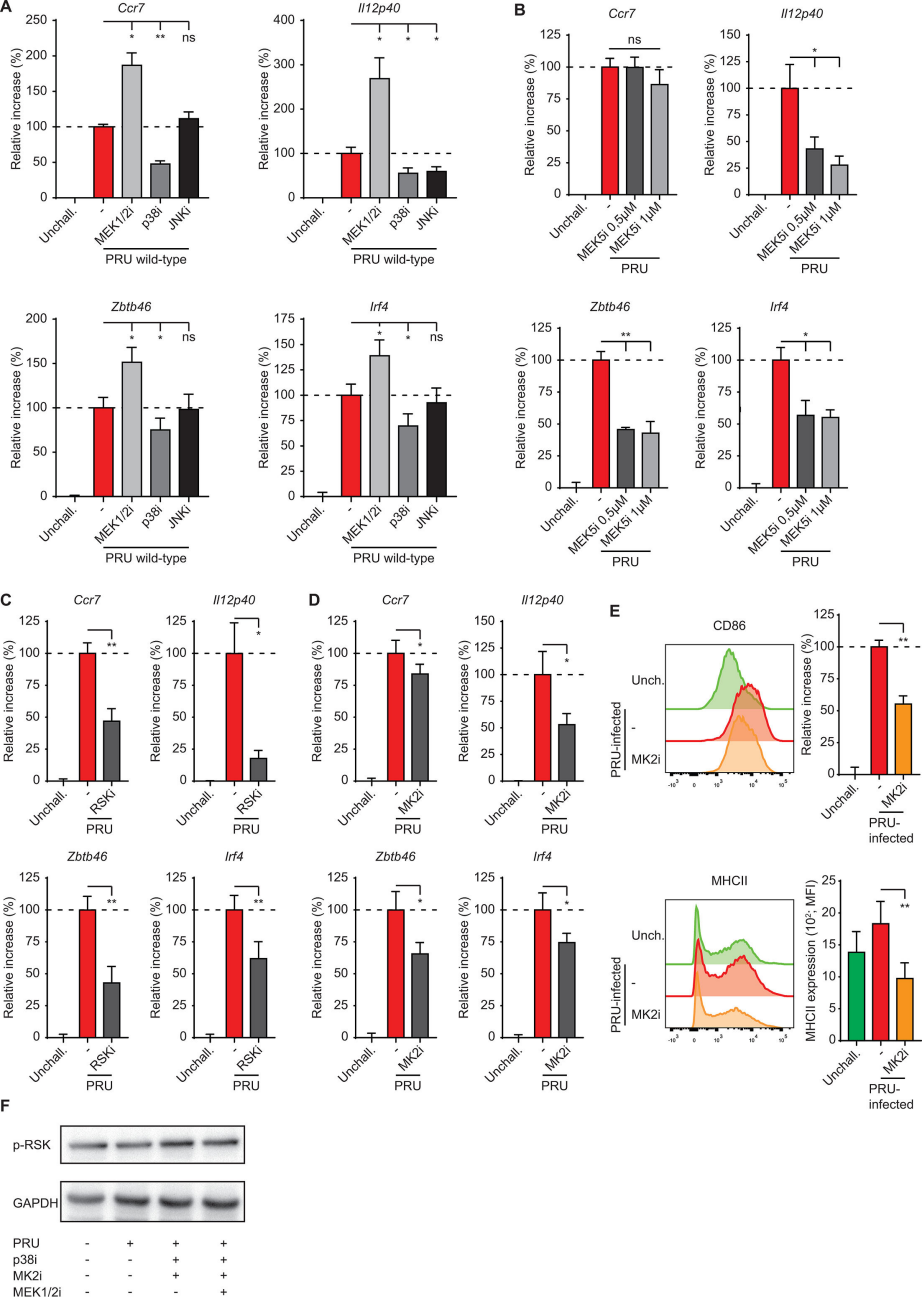
Migratory activation in *T. gondii*-infected macrophages involves MAPK-associated kinases. (**A** and **B**) Quantitative PCR (qPCR) analyses of *Ccr7*, *Il12p40*, *Zbtb46*, and *Irf4* cDNA from BMDMs challenged for 18 h with GFP-expressing *T. gondii* type II wild-type tachyzoites with (**A**) Trametinib (MEK1/2i), BIRB 796 (p38i), or JNK-IN-8 (JNKi) treatments, or (**B**) BIX02189 (MEK5i) treatment. Displayed is the increase in expression relative to untreated unchallenged (unchall., 0%) and wild-type (100%) challenged conditions [mean + SEM, *n* = 4 (**A**) and (**B**)]. (**C** and **D**) qPCR analyses of *Ccr7*, *Il12p40*, *Zbtb46*, and *Irf4* cDNA from BMDMs challenged for 18 h with GFP-expressing *T. gondii* type II wild-type tachyzoites with or without BRD7389 (RSKi; **C**) or MK2-IN-1 (MK2i; **D**) treatment. Displayed as in (**A**), *n* = 4 (**C**) or 3 (**D**). (**E**) Flow cytometric analysis of anti-CD86 and MHCII staining on BMDMs challenged for 18 h with freshly egressed GFP-expressing *T. gondii* type II wild-type tachyzoites (PRU; MOI 1), with or without MK2-IN-1 (MK2i) treatment, or left unchallenged. Infected (GFP^+^) cells were analyzed for challenged conditions. The bar graph displays the increase in expression, as in (**A**), *n* = 4. (**F**) Western blot analysis of p-RSK (S380/386) expression in lysates of BMDMs challenged for 5 h with wild-type *T. gondii* type II tachyzoites (PRU, MOI 3) in the presence of indicated inhibitors. Representative of two experiments. Statistical comparisons were made with ANOVA and Dunnett’s post-hoc tests (**A**), paired *t* test (**C–E**), and Spearman correlation (B; *P ≤ 0.05, **P ≤ 0.01, ***P ≤ 0.001, and ns *P* > 0.05).

Because MAPK signaling is strongly linked to cell motility and chemotaxis, we evaluated the impact of pharmacological inhibition of principal MAPK pathways (ERK1/2, ERK5, p38, and JNK) on the DC-like migratory activation of macrophages. The inhibition of p38 MAPK significantly reduced the expression of *Ccr7*, *Il12p40*, *Zbtb46*, and *Irf4* in PRU-challenged BMDMs (Fig. 3A), while MEK5 (ERK5) inhibition reduced *Il12p40*, *Zbtb46*, and *Irf4* expression (Fig. 3B). Notably, the transcriptional inductions were conversely amplified by MEK1/2 (ERK1/2) inhibition (Fig. 3A), in a GRA24-independent manner (Fig. S2A) and in line with reported inhibitory effects of ERK1/2 on IL-12 production (42). Yet, inhibition of Ca^2+^/calmodulin-dependent activation of MEK1/2 or ERK1/2 dimerization did not recapitulate this amplification (Fig. S2B). Consistent with the above and Δ*gra24* data (Fig. 2C), p38 inhibition abolished chemotaxis, which was maintained upon MEK1/2 inhibition (Fig. S2C). Jointly, the findings implicate p38 MAPK signaling in the GRA24-driven chemotactic responses of macrophages.

Next, we tested the impact of MAPK-activated kinases ribosomal S6 kinase (RSK) and MK2, two crucial downstream effectors of MAPK signaling (43). Inhibition of RSK nearly abolished the induction of *Il12p40* and significantly reduced the expression of *Ccr7*, *Zbtb46*, and *Irf4* in wild-type PRU-challenged BMDMs (Fig. 3C). Inhibition of MK2 also reduced *Il12p40*, *Ccr7*, *Zbtb46*, and *Irf4* expression in *T. gondii*-challenged BMDMs (Fig. 3D) and reduced MHCII and CD86 expression on *T. gondii*-infected BMDMs (Fig. 3E). In Bone marrow-derived dendritic cells (BMDCs) and BMDMs, RSK can be activated by ERK1/2 and, independently, p38-MK2 signaling (44). To confirm that RSK was indeed activated via p38-MK2 or ERK, we evaluated the RSK phosphorylation at Ser380/386. We found that p-RSK was readily detectable in unchallenged BMDMs, which was maintained in cells challenged with wild-type (PRU) or GRA24-deficient tachyzoites (Fig. S2D and E). However, p-RSK levels were not notably reduced by combinations of p38/MK2 or p38, MK2, and MEK1/2 inhibition in PRU-challenged BMDMs (Fig. 3F), making its activation mechanism elusive. Finally, because *Ccr7* expression can also be regulated by AP-1 (Fos/Jun) and ETS PU.1 transcription factors, which in turn are regulated by p38 MAPK (45, 46), we applied pharmacological inhibition. AP-1 and PU.1 inhibition did not impact the expression of *Ccr7*, *Zbtb46*, and *Irf4*, while the expression of *Il12p40* was significantly down- and upregulated, respectively (Fig. S3A and B). We conclude that, in *T. gondii*-infected BMDMs, p38-MK2 and RSK contribute to the induction of CCR7-dependent chemotaxis and DC-associated transcription factors, without across-the-board measurable involvement of AP-1 or PU.1.

### Additive effects of GRA15–NF-κB and GRA24–p38 signaling on the migratory activation of macrophages, with contributions by GRA16/18 and counteracting effects by TEEGR

**Fig 4:**
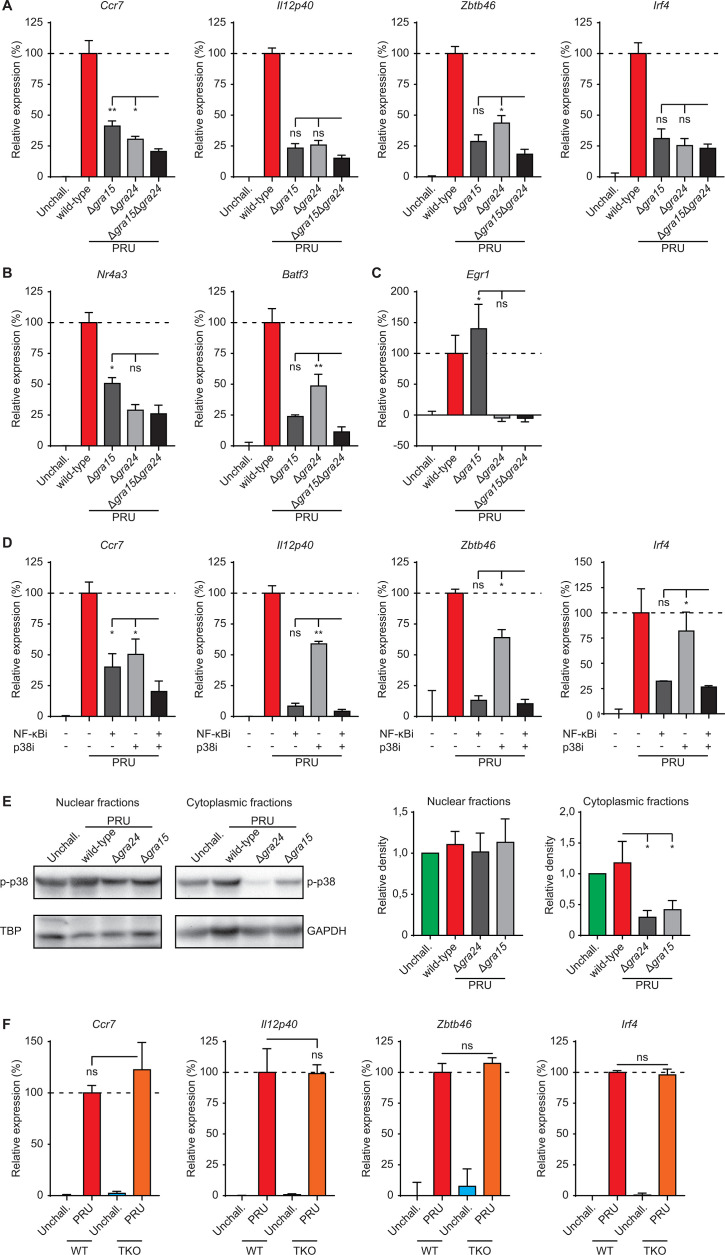
Effects of GRA15/24 double deficiency, NF-κB/p38 MAK inhibition, and canonical NF-κB signaling on the activation of *T. gondii*-infected macrophages (**A**), (**B**), and (**C**) quantitative PCR (qPCR) analyses of (**A**) *Ccr7*, *Il12p40*, *Zbtb46*, *Irf4*, (**B**) *Nr4a3*, *Batf3*, and (**C**) *Egr1* cDNA from BMDMs challenged with *T. gondii* type II PRU (wild-type), GRA15 (Δ*gra15*), GRA24 (Δ*gra24*), or GRA15/24 double mutant (Δ*gra15*Δ*gra24*) tachyzoites (18 h, MOI 2). Displayed is the increase in expression relative to untreated unchallenged (unchall., 0%) and wild-type (100%) challenged conditions (mean + SEM, *n* = 4). (**D**) qPCR analyses of *Ccr7*, *Il12p40*, *Zbtb46*, and *Irf4* cDNA from BMDMs challenged for 18 h with *T. gondii* type II wild-type tachyzoites in the presence of NFκB inhibitor (JSH-23 and NFκBi), p38 inhibitor (BIRB 796 and p38i), or combined treatment. Displayed is the increase in expression relative to untreated unchallenged (0%) and wild-type (100%) challenged conditions (mean + SEM; *n* = 3). (**E**) Western blot analysis of p-p38 (Thr180/Tyr182) expression in nucleus- and cytoplasm-enriched fractions of BMDMs challenged with PRU (wild-type), Δ*gra24*, or Δ*gra24* tachyzoites (5 h, MOI 3). Bar graphs display the relative density of p-p38 signal relative to TATA-binding protein (TBP) or GAPDH signals (mean + SEM; *n* = 4). (**F**) qPCR analyses of *Ccr7*, *Il12p40*, *Zbtb46*, and *Irf4* cDNA from wild-type BMDMs (WT) or Myd88^−/−^ Ticam^−/−^ Mavs^−/−^ BMDMs triple knock out (TKO) challenged for 18 h with *T. gondii* type II wild-type tachyzoites (MOI 2). Displayed is the increase in expression relative to wild-type (100%; mean + SEM; *n* = 3). Statistical comparisons were made with ANOVA and Dunnett’s post-hoc tests (*P ≤ 0.05, **P ≤ 0.01, ***P ≤ 0.001, and ns *P* > 0.05).

Because macrophages infected with either GRA15 or GRA24 single knockout parasites had reduced but not abolished *Ccr7* expression (Fig. 1 and 2), we hypothesized that collective effects were in play. BMDMs challenged with a GRA15/24 double mutant (Δ*gra15*Δ*gra24*) had significantly reduced *Ccr7* expression compared with Δ*gra15-* and Δ*gra24-*challenged BMDMs (Fig. 4A). Expression levels of *Il12p40*, *Zbtb46*, *Irf4*, *Nr4a3*, and *Batf3* were, however, not significantly further reduced by double-deficiency of GRA15/24 (Fig. 4A and B). Consistent with its GRA24 dependency, *Egr1* expression was abolished in PRUΔ*gra24-* and Δ*gra15*Δ*gra24*-challenged BMDMs, while GRA15-deficiency significantly elevated mRNA levels of *Egr1* (Fig. 4C; Fig. S1F). Next, we assessed the implications of NF-κB (GRA15) and p38 (GRA24) signaling by combined pharmacological inhibition. Individually, inhibitors partially inhibited *Ccr7*, *Il12p40*, *Zbtb46*, and *Irf4* expression in PRU-challenged macrophages with an overall superior effect upon NF-κB inhibition, while combined inhibition of NF-κB and p38, expectedly, mirrored the effects of challenge with Δ*gra15*Δ*gra24* parasites (Fig. 4D).

GRA15 activates NF-κB through tumor necrosis factor receptor-associated factors (TRAFs) (47). In turn, TRAF6 may also mediate p38 activation in cells (48). Consistent with this idea, western blotting revealed significantly lowered cytoplasmic p-p38 levels in BMDMs challenged with Δ*gra24* and, interestingly, also with Δ*gra15* parasites (Fig. 4E), indicating an impact of GRA15 on p-p38. Finally, to determine the contribution of NF-κB activation via pattern recognition receptors (PRRs), we challenged macrophages derived from Myd88^−/−^ Ticam^−/−^ Mavs^−/−^ mice (49). Interestingly, the responses of *T. gondii*-challenged mutant BMDMs approximated the responses by wild-type BMDMs (Fig. 4F), indicating a minor or non-significant contribution of NF-κB activation via PRRs to this phenotype. In sharp contrast, *Il12p35/p40* responses to LPS were undetectable in Myd88^−/−^ Ticam^−/−^ Mavs^−/−^ BMDMs (Fig. S4). Jointly, the data show that signaling linked to effectors GRA15 and GRA24 likely co-operate in the migratory activation and the DC-like transcriptional impact on macrophages via NF-κB signaling and p38 MAPK signaling, including a cross-regulation between the two pathways (50, 51). Yet, the intriguing finding that the effects on *Ccr7* were strongly reduced (~80%), but not strictly abolished, by GRA15/24 double-deficiency or by combined pharmacological inhibition motivated a further exploration of additional putative effectors.

Having established that NF-κB signaling contributes to the upregulation of *Ccr7*, *Il12p40*, and DC-associated transcription factors with pro-migratory effects, we investigated the effectors TEEGR, GRA16, and GRA18 because of their association with NF-κB signaling (52–54). Contrasting with the effects of GRA15 deficiency, BMDMs challenged with TEEGR-deficient type II tachyzoites (PRUΔ*teegr*) consistently expressed significantly higher levels of *Ccr7*, *Il12p40*, and *Zbtb46* compared with wild-type-challenged macrophages (Fig. S5A). In contrast, GRA16- (PRUΔ*gra16*) or GRA18-deficient (PRUΔ*gra18*) mutants presented a reduced induction of *Ccr7*, *Il12p40*, and *Zbtb46/Irf4* (Fig. S5B through D). Together, the data indicate that parasite effector TEEGR counteracts pro-migratory signaling, likely by NF-κB repression (52), while GRA16 and GRA18 promote signaling consistent with pro-migratory activation of infected macrophages.

### Selective impact of GRA28 on chromatin accessibility at the *Ccr7* locus and other gene loci implicated in the activation of macrophage migration

**Fig 5:**
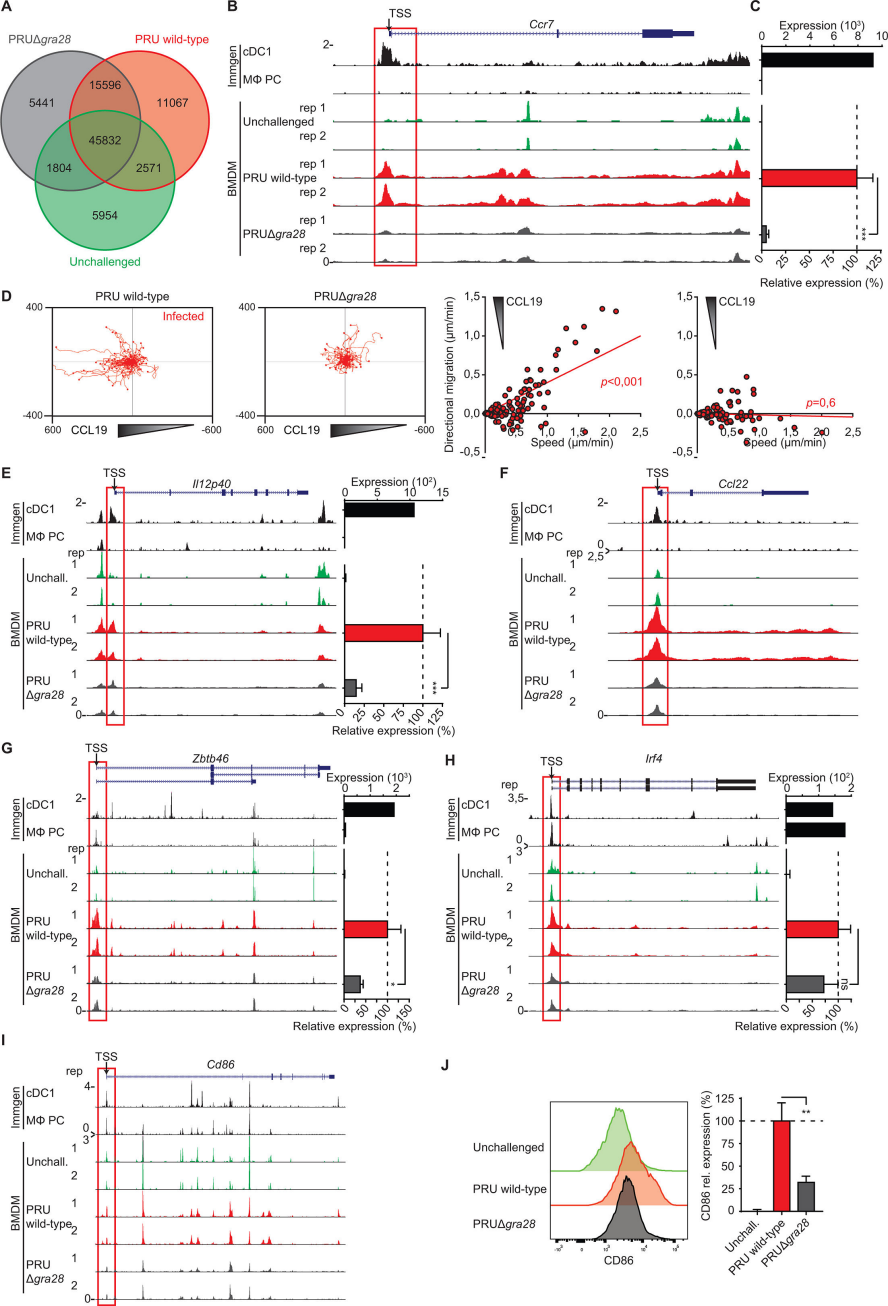
Chromatin accessibility and phenotypes of macrophages challenged with wild-type and GRA28-deficient *T. gondii* tachyzoites. (**A**) Venn diagram shows numbers of identified ATAC-seq peaks in unchallenged BMDMs and BMDMs challenged for 18 h with *T. gondii* type II wild-type or GRA28-deficient (Δ*gra28*) tachyzoites (PRU; MOI 2). (**B**) Genome tracks show ATAC-seq peak intensities, normalized to uniquely aligned reads (y-axis), around the promoter of *Ccr7* gene. For BMDMs, ATAC-seq signal is from two separate biological replicates per condition. Upper tracks show peak signals from dendritic cells (cDC1) and peritoneal cavity macrophages (MΦ PC) extracted from ImmGen publicly available data set. Red outline indicates the region of interest near the transcription start site (TSS). (**C**) Upper bar graph shows *Ccr7* expression by cDC1 and MΦ PC, quantified by RNA-seq (ImmGen publicly available data set). Lower bar graph shows qPCR analysis of *Ccr7* cDNA from BMDMs challenged for 18 h with *T. gondii* type II wild-type or GRA28-deficient (Δ*gra28*) tachyzoites (PRU; MOI 2) or left unchallenged. The increase in expression relative to unchallenged (unchall., 0%) and wild-type (100%) challenged conditions is displayed (mean + SEM, *n* = 5). (**D**) Motility plots depict the displacement of BMDMs challenged with CMTMR-stained *T. gondii* type II wild-type and GRA28-deficient (Δ*gra28*) tachyzoites (PRU; MOI 1) in a CCL19 gradient, as detailed in Materials and methods (scale indicates µm). For each condition, directional migration (µm/min) toward the CCL19 source and speed (µm/min) of individual cells is displayed in graphs, with linear regression lines. Infected cells (CMTMR^+^) were analyzed from three independent experiments. For each condition, *P*-values indicate the directional migration compared to hypothetical zero directionality (one-sample permutation test). (**E–I**) ATAC-seq genome tracks for (**E**) *Il12p40*, (**F**) *Ccl22*, (**G**) *Zbtb46*, (**H**) *Irf4*, and (**I**) *CD86* in cDC1 and MΦ PC as in (**B**) and transcriptional analyses as in (**C**). (**J**) Flow cytometric analysis of anti-CD86 staining on BMDMs challenged for 18 h with CFSE-stained *T. gondii* type II wild-type tachyzoites (PRU; MOI 1). Infected (CFSE^+^) cells were analyzed for challenged conditions. The bar graph displays expression related to wild type (mean + SEM, *n* = 4). Statistical comparisons were made with ANOVA and Dunnett’s post-hoc tests (C, E, G, H, J, *P ≤ 0.05, **P ≤ 0.01, **P ≤ 0.001, and ns *P* > 0.05).

Gene expression is linked to chromatin accessibility, which is modulated by chromatin remodeling complexes, such as SWI/SNF (55). Analyses of publicly available data sets (56) using Assay for Transposase-Accessible Chromatin with sequencing (ATAC-seq) revealed accessible chromatin around the promoters of *Ccr7*, *Il12p40*, and *Zbtb46* genes in different types of DCs but not in macrophages (Fig. S6A). We previously showed that GRA28 (type I) interacts with the SWI/SNF complex and binds to chromatin (21). Together, this motivated an assessment of chromatin accessibility for genes linked to the migratory activation of BMDMs using ATAC-seq, in the presence or absence of GRA28 (type II). Our analysis detected 26,663 open chromatin peaks uniquely found in BMDMs challenged with wild-type (PRU) tachyzoites compared with unchallenged BMDMs (Fig. 5A; Fig. S6B). Furthermore, among these 26,663 peaks, 11,067 peaks were not detected in PRUΔ*gra28*-challenged BMDMs, underlying the involvement of GRA28 in chromatin accessibility (Fig. 5A). Importantly, around the transcription start site of *Ccr7*, we found a notable increase in accessible chromatin in wild-type PRU-infected BMDMs compared with unchallenged BMDMs, which is a typical feature of DCs (Fig. 5B). Supporting the idea that GRA28 drives chromatin accessibility, we observed a reduction in accessible chromatin in PRUΔ*gra28*-infected BMDMs (Fig. 5B). In sharp contrast, increases in chromatin accessibility were not detected around promoters of *Ccr2*, *Ccr5*, and *Cx3cr1* genes, coding for other chemokine receptors in phagocytes (Fig. S7A). In line with ATAC-seq data, GRA28-deficiency (PRUΔ*gra28*) nearly abolished the induction of *Ccr7*, determined by RT-qPCR (Fig. 5C). Finally, chemotaxis to CCL19 was undetectable in BMDMs challenged with PRUΔ*gra28* strain (Fig. 5D), functionally corroborating the findings.

Furthermore, chromatin accessibility was GRA28-dependently elevated around the promoter of *Il12p40* (Fig. 5E), and the promoter of *Ccl22*, a known target chemokine for GRA28 (57) (Fig. 5F). In contrast, the accessibility was not elevated for genes encoding cytokines *Ccl24*, *Tnf*, and *Il1a* (Fig. S7B). For DC-associated transcription factors, analyses showed GRA28-dependent elevated chromatin accessibility around the promoters of *Zbtb46* (Fig. 5G) and *Batf3* (Fig. S7C), while accessibility changes were less evident for *Irf4* (Fig. 5H) and *Nr4a3* (Fig. S7C). Finally, ATAC-seq detected minor changes for *Cd86* (Fig. 5I), and we observed a decreased expression of CD86 in PRUΔ*gra28*-infected BMDMs compared with wild-type-infected BMDMs, determined by flow cytometry (Fig. 5J). We conclude that GRA28 selectively elevates chromatin accessibility around the promoters of *Ccr7*, *Il12b*, *Ccl22*, and genes encoding DC-associated transcription factors, thereby impacting their transcription in *T. gondii*-infected BMDMs.

### Impact of GRA15/24 double deletion on *T. gondii* dissemination in mice and on the phenotypes of human macrophages/monocytes

**Fig 6:**
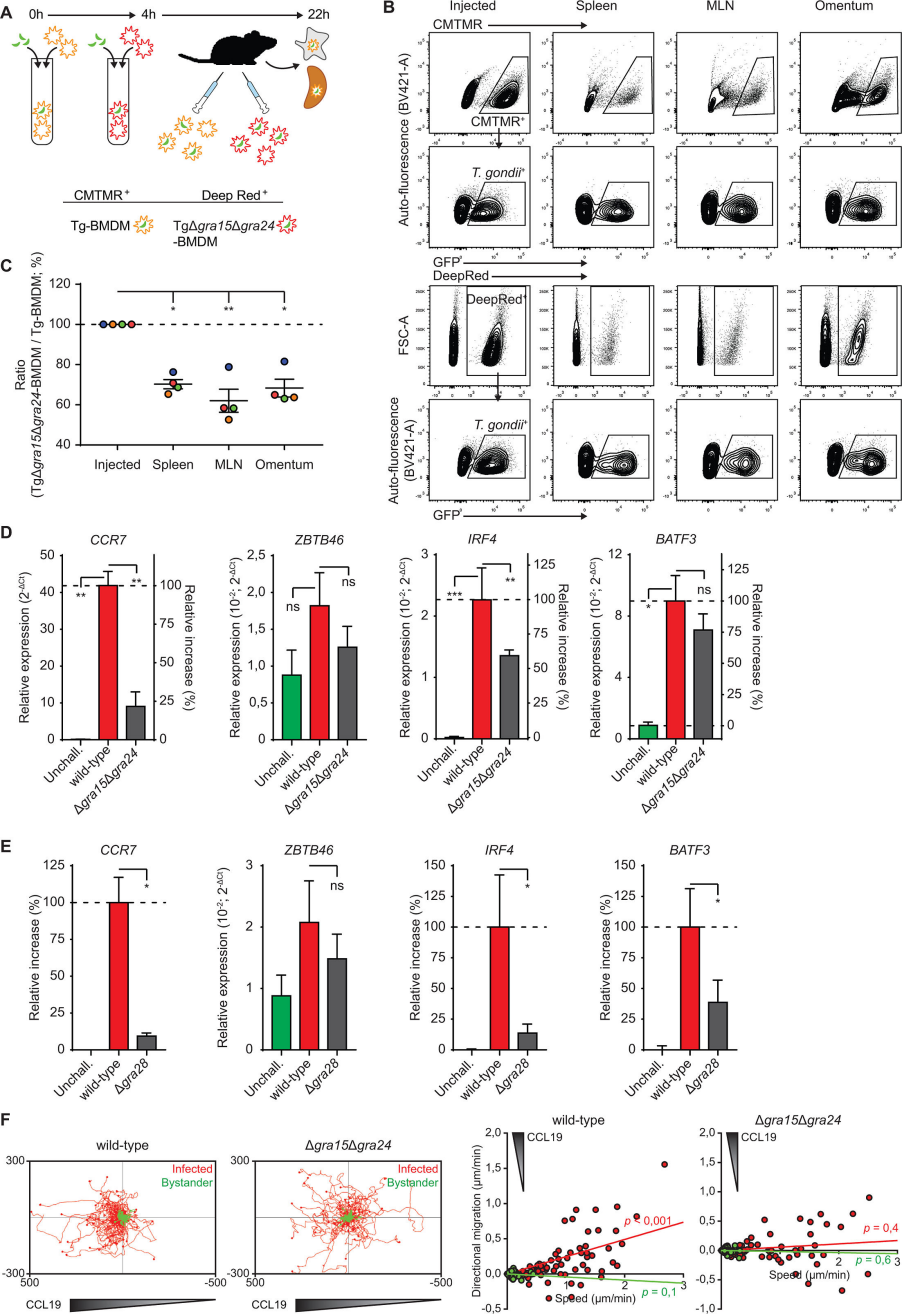
Impact of GRA15/24 on *T. gondii* dissemination in mice and on the phenotypes of human macrophages and monocytes. (**A**) Illustration of experimental setup and conditions for co-adoptive transfers of BMDMs challenged with *T. gondii* (PRU) wild-type (Tg-BMDM) or GRA15/24 double mutant (TgΔ*gra15*Δ*gra24*-BMDM) and pre-labeled with CMTMR or CellTracker Deep Red dyes, respectively. (**B**) Contour plots show a typical gating strategy for flow cytometric detection of pre-labeled and *T. gondii* parasitized BMDMs (CMTMR^+^/Deep red^+^ and GFP^+^) as injected intraperitoneally and extracted from spleen, MLN, and omentum 18 h post-inoculation, as detailed under Materials and methods. Single (CD11c^+^) BMDMs were pre-gated as shown in Fig. S8A. (**C**) Flow cytometric analysis of wild-type- or Δ*gra15*Δ*gra24*-challenged BMDMs in the spleen, MLNs, and omentum 18 h post-inoculation. Data are presented as the change in ratio between detected challenged Deep red^+^GFP^+^ (Δ*gra15*Δ*gra24*) cells and CMTMR^+^GFP^+^ (wild-type) cells related to the inoculated ratio (normalized to 100%). Mean ratio change ±SE and individual mice (*n* = 4) are displayed. (**D** and **E**) qPCR analyses of *Ccr7*, *Il12p40*, *Zbtb46*, and *Irf4* cDNA from human monocyte-derived macrophages (mo-macs) challenged with *T. gondii* type II PRU (wild type), (**D**) Δ*gra15*Δ*gra24*, or (**E**) Δ*gra28* tachyzoites (18 h, MOI 2). Displayed is a relative expression (2^−ΔCt^) or the relative and increase in expression relative to untreated unchallenged (unchall., 0%) and wild-type (100%) challenged conditions (mean + SEM, *n* = 4). (**F**) Motility plots depict the displacement of mo-macs challenged with wild-type and Δ*gra15*Δ*gra24* tachyzoites (14 h MOI 1) in a CCL19 gradient as detailed in Materials and methods (scale indicates µm; *n* = 3). For each condition, directional migration (µm/min) toward the CCL19 source and speed (µm/min) of individual cells is displayed in graphs, with linear regression lines. Infected cells (GFP^+^, red) were analyzed. For each condition, *P*-values indicate the directional migration compared to hypothetical zero directionality (one-sample permutation test). Statistical comparisons were made with weighted least square and Dunnett’s post-hoc tests (**C**) or ANOVA and Dunnett’s post-hoc tests (D, E *P ≤ 0.05, **P ≤ 0.01, **P ≤ 0.001, and ns *P* > 0.05).

We previously established a role for GRA28 in the migration of infected macrophages to secondary organs (21). Given the collective contributions of GRA15/24/28 to gene expression leading to pro-migratory activation of macrophages *in vitro*, we addressed the impact of GRA15/24 signaling *in vivo* on parasite dissemination. Equivalent numbers of pre-labeled BMDMs challenged with wild type or Δ*gra15*Δ*gra24* parasites were adoptively transferred i.p. to mice in competition assays (Fig. 6A). Fourteen to 18 h post-inoculation, organs were harvested, and cells were characterized by flow cytometry (Fig. 6B; Fig. S8A). Importantly, BMDMs challenged with *Δgra15Δgra24 T. gondii* migrated at a relatively lower frequency to the omentum, mesenteric lymph nodes (MLNs), and spleen, compared with BMDMs challenged with wild-type *T. gondii* (Fig. 6C), showing an implication of GRA15/24 in the migration of parasitized BMDMs in mice.

Furthermore, we extended our key findings in murine cells by challenging human peripheral blood monocytes or monocyte-derived macrophages (mo-macs) with two separate type II *T. gondii* lines (PRU or ME49-PTG). *T. gondii*-challenged cells expressed elevated levels of *CCR7*, *ZBTB46*, *IRF4*, and *BATF3* compared with their unchallenged counterparts (Fig. 6D; Fig. S8B). Similar to murine BMDMs, challenge with GRA15/24-deficient tachyzoites yielded decreased expressions (Fig. 6D), in line with effects of GRA28 deficiency (Fig. 6E). Finally, we confirmed abolished chemotaxis in a CCL19 gradient by mo-macs challenged with GRA15/24-deficient parasites (Fig. 6F), with maintained hypermotility (Fig. S8C). Jointly, we conclude that GRA15/24/28 potentiates the migration of infected macrophages to secondary organs in mice and induces pro-migratory transcriptional activation and CCR7-driven chemotaxis in human macrophages and monocytes.

## DISCUSSION

**Fig 7:**
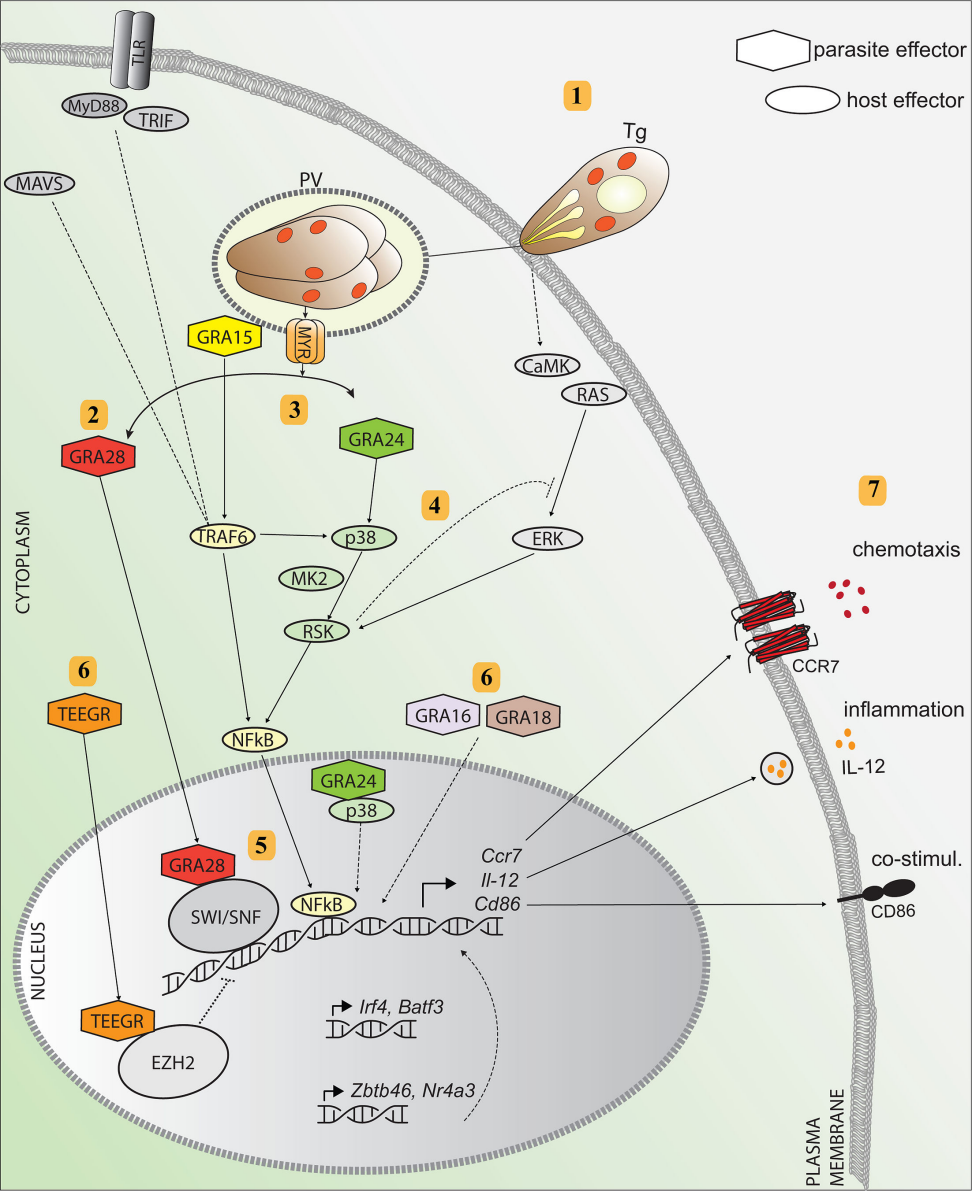
Hypothetical model for the migratory activation of parasitized macrophages by *Toxoplasma*. (1) *T. gondii* actively invades the macrophage and forms a PV where it replicates and secretes effector proteins from secretory organelles (dense granules) into the host cell cytosol via the MYR1 translocon. (2) The effector GRA28 is secreted into the host cell cytosol MYR1 dependently and locates to the nucleus where it complexes with chromatin remodelers (SWI/SNF) to open up chromatin. (3) GRA15 is secreted MYR1 independently and interacts with TRAF6 to activate NF-κB, which locates to the nucleus. In parasitized macrophages, NF-κB activation occurs independently of TLR/MyD88/TRIF/MAVS signaling. (4) MYR1-dependent secretion of GRA24 activates p38 signaling and potentiates NF-κB activation via RSK and MK-2. RSK is also regulated via RAS-ERK MAPK pathway, which becomes activated via CaMK upon *T. gondii* infection (16). The GRA24/p38 complex can also travel to the nucleus. (5) GRA28-mediated increased chromatin accessibility facilitate GRA15/24/p38-driven transcription via NF-κB at the *Ccr7*, *Il12*, *and Cd86* gene loci. (6) GRA16 and GRA18 contribute to macrophage activation by unknown mechanisms. In contrast, the effector TEEGR counteracts activation, presumably through interaction with the transcriptional repressor EZH2. The elevated transcription of DC-related transcription factors (*Zbtb46*, *Irf4*, *Nr4a3*, and *Batf3*) also drives expression of *Ccr7*, *Il12*, *and Cd86*. (7) Altered signaling results in elevated expression of CCR7 with chemotactic responses, pro-inflammatory IL-12, and CD86 expression. Color-coded hexagonal shapes represent parasite effectors, and oval shapes represent corresponding host effectors and signaling pathways. Dashed lines represent hypothetical signaling or signaling not addressed here.

Here, we addressed how *T. gondii* orchestrates the migratory activation of macrophages that promote systemic parasite dissemination. We demonstrate that a set of secreted GRA proteins regulates the chemotactic and pro-inflammatory activation of parasitized macrophages. First, we identify novel functions for GRA15 and GRA24 in promoting CCR7-mediated chemotactic responses. Second, we show that GRA15 and GRA24 cooperate by acting on NF-κB and p38 MAPK signaling pathways, respectively, with minor contributions by GRA16 and GRA18 and counteracting effects by TEEGR to the migratory and pro-inflammatory responses of parasitized macrophages. Third, we report that GRA28 elevates chromatin accessibility at the *Ccr7* locus and other loci associated with pro-migratory activation of macrophages. Finally, we show that GRA15/24 impact the systemic transport of *T. gondii* in mice, similar to GRA28 (21). We propose a model for how the concerted action of GRA effectors activates parasitized macrophages (Fig. 7).

We report a role for GRA15 in the migratory activation and CCR7-driven chemotaxis of parasitized macrophages via its activation of NF-κB signaling (30). The findings have a direct bearing on the *Trojan horse* mechanism for *T. gondii* dissemination also because GRA15 impacts ICAM-1 expression (58), and adhesion molecules regulate the motility and migration modes of macrophages in tissues (59). In line with this, we recently reported that DCs infected with GRA15-deficient *T. gondii* exhibit reduced transmigration across endothelium (58). However, GRA15 deficiency reduced, but did not strictly abolish, transcriptional activations and the migratory phenotypes of macrophages and DCs. Moreover, the migratory phenotypes are present in the type I RH line, which lacks a functional GRA15 (60). Thus, while GRA15 likely contributes to the reported enhanced transmigration frequencies of type II-infected DCs over type I-infected DCs (12), additional effectors are in play in a strain/genotype-dependent manner.

We demonstrate a role for GRA24 in the migratory activation of infected macrophages, mediated via p38 MAPK signaling. In type II *T. gondii*-infected macrophages, this response was prominent and co-operated with GRA15 in eliciting chemotactic and pro-inflammatory activation. Consistent with this, GRA15 and GRA24 were recently reported to synergistically promote pro-inflammatory cytokine expression in type II strains (61). Interesting findings in this context are the impact of GRA15 on p38 phosphorylation and the p38-linked activation of RSK, jointly advocating for cross-regulation between NF-κB and p38 MAPK signaling. Consequently, a GRA15/24 double mutant dramatically reduced the migratory activation of macrophages *in vitro* and *in vivo*, corroborating the importance of these two parasite effectors. Similarly, combined inhibition of NF-κB and p38 MAPK signaling mirrored these effects. Yet, in neither case was the phenotype totally abrogated, indicating the implication of additional effectors or signaling. Interestingly, the dense granule protein TEEGR acted as a down-modulator of *Ccr7*, *Il12p40*, and other responses, presumably related to its negative regulation of NF-κB (52). In contrast, GRA16 and GRA18 positively contributed to the phenotype, however, to a lesser extent than GRA15 or GRA24. Along these lines, in type I (RH) *T. gondii*-infected macrophages, we found that the responses, specifically CCR7 chemotaxis, were less dependent on GRA24 (21). One explanation might be that, because the type I RH strain lacks a functional GRA15 protein that activates NF-κB responses (30), NF-κB activation is mediated by other GRA or non-GRA effectors, and the balance between NF-κB activating and counteracting effectors is shifted (52, 62, 63). Finally, the comparable induction of *Ccr7*, *Il12p40*, *Zbtb46*, and *Irf4* expression observed in both wild-type and Myd88^−/−^ Ticam^−/−^ Mavs^−/−^ macrophages (49) indicates that, upon *T. gondii* infection, activation of NF-κB signaling predominantly occurs in a manner that is independent of the MyD88/TRIF/IPS-1-mediated pathways for NF-κB activation. Instead, the mounting data indicate that the activation of NF-κB for this phenotype occurs via TRAFs (30, 47).

We demonstrate that GRA28 selectively elevates chromatin accessibility at the *Ccr7* gene locus. This effect, in conjunction with GRA28’s interaction with the chromatin remodeler SWI/SNF (21), establishes a central role for GRA28 in the migratory activation of macrophages by both type I and II *T. gondii* strains. We propose a mechanistic framework in which GRA28 interacts with chromatin remodelers to fine-tune the transcriptional regulation directed by GRA15—NF-κB and GRA24—p38 MAPK, targeting the expression of *Ccr7*, *Il12p40*, and *Cd86* (Fig. 7). Further corroborating this model, the *Ccr7* promotor contains four putative NF-κB-binding sites and a single putative AP-1/c-Fos-binding site (64). Pharmacological inhibitor data indicate that NF-κB, but not AP-1 or PU.1, is the major transcription factor in play. Jointly, the findings establish a central role for the GRA15/24/28 triad in the migratory activation of macrophages, supporting previous observations that genotype-specific parasite effectors influence *T. gondii* dissemination by parasitized leukocytes (12). GRA28 selectively enhanced chromatin accessibility at the *Ccr7*, *Il12p40*, and *Ccl22* gene loci, without a detectable enhancement at gene loci of other important cytokines, chemokines, or chemokine receptors. Thus, the molecular determinants guiding the site specificity of GRA28 or GRA28/SWI/SNF complexes at these loci warrant further investigation.

Why does *T. gondii* express and secrete multiple effectors that co-operate in the migratory activation of phagocytes? *T. gondii* infects and replicates within different types of immune cells in a broad array of vertebrate species. Seeking latency in warm-blooded vertebrates to assure later transmission to the feline definitive host necessitates dissemination to various organs, especially the brain, which must be executed across a range of host species and immune cell types. Moreover, different anatomical sites and temporal stages of infection might necessitate distinct migratory behaviors such as reverse transmigration, afferent migration, and motility in tissues. Thus, polymorphisms and functional redundancy in effector proteins may enable *T. gondii* to adapt to both inter-host and intra-host diversity. Our current understanding points toward a nuanced activation of different phagocytic cell types. Specifically, while DCs are readily chemotactically activated (22, 23), macrophages exhibit relative resilience against such activation, as demonstrated by their lack of CCR7 upregulation or chemotaxis upon PRR stimulation (21, 65). GRA28 appears instrumental in overcoming this resilience by mediating chromatin accessibility at the *Ccr7* locus, thus enabling NF-κB-induced activation where it would otherwise not occur.

GRA15/24/28 target complementary host pathways eventually leading to CCR7 expression and migratory activation. It is reasonable to speculate that such system redundancy ensures the robustness of leukocyte hypermigration, a hypothesis supported by existing literature (66). Furthermore, the diversity in effector molecules could potentially aid in evading host immune recognition, thereby thwarting efforts at neutralization. On the other hand, hypermigration of phagocytes might also increase contact between immune cells, for example, with the T cell compartment (67), potentially leading to increased immune control, albeit, without hindering chronic infection. Lastly, the observed counteractive interactions among various GRA and ROP proteins, such as GRA15 vs TEEGR for NF-κB activation or ROP16 vs GRA28 for CCR7 regulation (21), hint at an additional layer of complexity, possibly functioning as a fine-tuning mechanism or as on-off switches. Therefore, it can be postulated that effector diversification serves as a flexible strategy for the parasite, allowing for both stringent regulation and evolutionary adaptability across multiple hosts.

Microbial pathogens have developed elaborated strategies to thrive inside phagocytes and colonize their hosts (68–70). For example, recent findings show that *Mycobacterium tuberculosis* manipulates alveolar macrophage trafficking for rapid localization to the lung interstitium (71). Furthermore, *Leishmania* and *Salmonella typhimurium* inhibit macrophage and DC motility (72, 73), while coccidian parasites elevate the migration of phagocytes (13). However, a detailed molecular understanding of how pathogens orchestrate the hijacking of complex cellular processes, such as host cell migration, is yet to come. The present findings provide a molecular framework delineating how *T. gondii* orchestrates the migratory activation of phagocytes, primarily, but not exclusively, via the concerted action of GRA15/24/28 targeting CCR7-mediated chemotaxis. Furthermore, MYR1 and GRA28 mutants have similar phenotypes (21). Because the type I strain RH expresses a non-functional form of GRA15 (30) and GRA24/28 are secreted MYR1-dependently, MYR1-deficiency in type I RH may phenotypically reflect a combined GRA15/24/28-deficiency in this respect. Yet, hypermotility is maintained in GRA15/24/28 mutants (21, 37). Indeed, in the hypermigratory responses of phagocytes (17), separate but partly overlapping signaling regulates GABA/voltage-dependent calcium channel (VDCC)-driven hypermotility and CCR7-driven chemotactic activation of parasitized phagocytes. Specifically, the rhoptry protein TgWIP modulates the host cell actin dynamics presumably via WAVE/arp2/3 complex (20), while ROP17 presumably acts on RhoGTPases (19) and via the MYR1 complex (21, 74). Also, Tg14-3-3-related sequestration of host 14-3-3 to the PV presumably impacts MAPK signaling (16, 18). However, the specific parasite-derived effector(s) activating motogenic GABAergic and VDCC-mediated signaling remain to be identified (13). Thus, the diversity and redundancy of polymorphic effectors also highlight the importance of distinguishing between *in vivo* fitness readouts and defined migratory phenotypes linked to dissemination when investigating effectors in *in vivo* screens (20, 75, 76). In summary, we propose that the joint activities of these effectors culminate in pro-migratory signaling within the infected phagocyte. Such coordinated action may not only facilitate the parasite’s dissemination within multiple hosts but could also confer advantages in evading host immune responses. The implications of this pro-migratory signaling state and its ultimate consequences for host-pathogen interactions merit further in-depth investigation.

## MATERIALS AND METHODS

Cell lines, mouse strains, and parasite strains are described in Table S1. Antibodies, chemicals, and kits are detailed in Table S2.

### Mouse cell culture

Cells from bone marrow of 6–10-week-old male or female wild-type or Myd88^−/−^ Ticam^−/−^ Mavs^−/−^ (49) C57BL/6 mice (see Table S1) were cultivated in RPMI 1640 (VWR) with 10% fetal bovine serum (FBS; HyClone), gentamicin (20  µg/mL; Sigma-Aldrich), and glutamine (2 mM), referred to as complete medium (CM), and supplemented with 20 ng/mL recombinant mouse GM-CSF or (when indicated) 10% L929 conditioned medium. Strongly adherent cells were harvested on days 6–8 as BMDMs. For PEMs, C57BL/6 mice were euthanized, and peritoneal lavage (10 mL PBS) was collected from the peritoneal cavity. After overnight culture in a complete medium, loosely and non-adherent cells were removed by repeated washing, and the adherent cells were used as PEM in experiments for RNA isolation.

### Human cell culture

Human CD14^+^ monocytes were isolated from peripheral blood mononuclear cells after density gradient centrifugation on Lymphoprep with CD14 MicroBeads from buffy coats obtained from healthy donors at the Karolinska University Hospital Blood Center and cultured in complete medium. Monocyte-derived macrophages (mo-macs) were generated from CD14^+^ monocytes through culture for 3–5 days in a complete medium supplemented with 20 ng/mL human recombinant GM-CSF.

Human foreskin fibroblasts HFF-1 were cultured in Dulbecco‘s modified Eagle’s medium, high glucose, (DMEM; VWR) with 10% FBS (HyClone), gentamicin (20 µg/mL; Sigma-Aldrich), glutamine (2 mM), and HEPES (0.01 M), referred to as DMEM.

### Parasite culture

*T. gondii* tachyzoites were maintained by serial 2-day passages in human foreskin fibroblast HFF-1 monolayers. Freshly egressed tachyzoites were used for all infections. The different strains used are listed in Table S1. All cell cultures used were periodically tested for mycoplasma and found to be negative.

### Infection challenges

Carry-over from routine *T. gondii* culture to experiments was minimized by repeated washing of the freshly egressed tachyzoites before challenge with live tachyzoites. For all quantitative PCR (qPCR), ATAC-seq, western blot experiments, BMDMs, human monocytes, mo-macs, and/or PEMs were challenged with freshly egressed *T. gondii* tachyzoites with indicated strains/lines at MOI 2 for 18 h, unless differently stated. LPS was used at a final concentration of 10 ng/mL. For flow cytometry assays, BMDMs were challenged for 18 h with GFP-expressing or CFSE-labeled *T. gondii* tachyzoites with the indicated strains (MOI 1). For chemotaxis experiments, BMDMs were challenged for 14 h with GFP-expressing or CFSE-labeled *T. gondii* tachyzoites at MOI of 1 before seeding in the chemotaxis chamber.

### Inhibitors

When indicated, cells were treated with inhibitors Trametinib (1 µM), BIRB 796 (10 µM), JNK-IN-8 (3 µM), BRD7389 (5 µM), MK2-IN-1 (5 µM), TPCA-1 (3 µM), SR 11302 (20 µM), T-5224 (2 µM), DEL-22379 (5 µM), DB2313 (1 µM), BIX02189 (0.5 or 1 µM), and/or JSH-23 (25 µM) vehicle (DMSO) initiated 1 h prior to challenge.

### Quantitative PCR

BMDMs, PEMs, and human monocytes and mo-macs were cultured with complete medium or challenged with freshly egressed *T. gondii* tachyzoites of the indicated strains and lysed in TRI Reagent (Sigma-Aldrich) or Lysis buffer (Jena Bioscience). Total RNA was extracted according to the manufacturer’s protocol using the Direct-zol RNA Miniprep (Zymo Research) or Total RNA Purification (Jena Bioscience) kits and reverse transcribed with Maxima H Minus Reverse Transcriptase (Thermo Fisher). Real-time qPCR was performed with SYBR green PCR master mix (KAPA biosystems) or HotStart 2× SYBR Green qPCR Master Mix (APExBIO Technology), specific forward and reverse primers at target-dependent concentrations (100–200 nM), and cDNA (10–30 ng) in a QuantStudio 5 System (Thermo Fisher) with ROX as a passive reference. qPCR results were analyzed using the ΔCq method relative to Importin-8 and TATA-binding protein as housekeeping genes and displayed as such or normalized to unchallenged (set to 0%) and wild-type *T. gondii*-challenged (set to 100%). Primers are listed in Table S3.

### Assay for transposase-accessible chromatin with sequencing

To measure chromatin accessibility, we performed ATAC-seq using ATAC-Seq Kit according to the manufacturer’s instructions. After challenging macrophages with *T. gondii*, we centrifuged 100,000 cells at 500 × *g* at 4°C for 5 min, removed the supernatant, and washed the cells once with 100 µL ice-cold 1 × PBS without disturbing the cell pellet. We next performed additional centrifugation at 500 × *g* at 4°C for 5 min. Afterward, we removed 1 × PBS and resuspended the cells in 100 µL ATAC lysis buffer which is immediately followed by centrifugation at 500 × *g* at 4°C for 10 min. After removing the supernatant, we incubated the nuclei in 50 µL Tagmentation Master Mix (25 µL 2 × Tagmentation buffer, 12 µL water, 10 µL Assembled Transposomes, 2 µL 10 × PBS, 0.5 µL 1% Digitonin, and 0.5 µL 10% Tween 20) at 37°C for 30 min using PCR machine with heated lid. Following the incubation, we added 250 µL DNA-binding buffer and 5 µL sodium acetate and performed column purification by centrifugation at 17,000 × *g* for 1 min. The column was washed once with 750 µL wash buffer and centrifuged at 17,000 × *g* for 1 min. Finally, we eluted tagmented DNA from the column using 35 µL DNA Purification Elution Buffer by centrifugation at 17,000 × *g* for 1 min.

### PCR amplification of tagmented DNA

Combine 33.5 µL previously isolated tagmented DNA with 10 µL 5 × Q5 Reaction buffer, 2.5 µL 25 µM i7 Indexed primer, 2.5 µL 25 µM i5 Indexed primer, 1 µL 10 mM dNTPs, and 0.5 µL 2 U/µL Q5 Polymerase. The reaction mixture was then incubated on a thermocycler with the following PCR parameters: 72°C for 5 min, 98°C for 30 s, 10 cycles of 98°C for 10 s, 63°C for 30 s, and 72°C for 1 min. ATAC-seq libraries were sequenced as 79 + 79 nt paired-end reads using NextSeq 550 (Illumina).

### Analysis of ATAC-seq data

ATAC-seq data set was aligned via Bowtie 2.2.5 (77) with parameters “--very-sensitive --no-unal --no-mixed --no-discordant -I 10× 700.” The alignment results were converted to bigWig signal tracks using BEDtools 2.27.1 and SAMtools (78) and normalized to uniquely aligned reads. The signals (log10 RPM with pseudo count 0.01) at transcription start site ±500 bp were also calculated for each sample and used for generating Pearson correlation co-efficiency. MACS2 (79) was then applied to call peaks with default parameters. Peaks present in both independent biological replicates were regarded as representative peaks for each condition and used for generating the Venn diagram.

### Flow cytometry

BMDMs were challenged as indicated and stained with anti-CD11c, CD11b, MHCII I-A/I-E, CD86, or isotype control antibodies and live/dead far red stain. Staining was performed on fixed (2% PFA) or live cells, blocked with anti-CD16/CD32 antibody, in FACS buffer (PBS/0.5% FBS/1 mM EDTA). Flow cytometry was performed on a BD LSRFortessa flow cytometer (BD Biosciences) and analyzed with FlowJo X (FlowJo LLC).

### Western blot

For western blotting, cells were challenged as indicated, harvested, washed with PBS, and then lysed directly in Laemmli buffer for whole-cell lysates. Proteins were separated using 10% SDS-PAGE, blotted onto a PVDF membrane, and blocked (10% BSA in TBS/0.5% Tween-20) followed by incubation with primary and secondary antibodies: anti-TATA-binding protein, anti-p-IκBα (Ser32/36), anti-p-RSK (Ser380/386), anti-p-p38 (Thr180/Tyr182) anti-mouse, anti-rabbit, or anti-rat IgG-HRP in 5% BSA/0.5% Tween-20 in TBS. Proteins were revealed by means of enhanced chemiluminescence (GE Healthcare) in a BioRad ChemiDoc XRS+. Densitometry analysis was performed using ImageJ (NIH, MD, USA). For display, the contrast of images was enhanced with the ImageJ “enhance contrast” feature without pixel saturation.

### Chemotaxis

BMDMs or human macrophages were challenged as indicated, washed, resuspended in complete medium (CM) with 1 mg/mL bovine collagen type I (Sigma), and seeded into uncoated ibiTreat μ-slide chemotaxis chambers (Ibidi, Martinsried, Germany). Collagen was allowed to polymerize for 30 min, and media, inhibitors, and 1.25 µg/mL murine (BMDMs) or human recombinant CCL19 (human macrophages) were added as indicated conform the manufacturer’s instructions (application note 23). Cells were then imaged every 5 min for 8 h (Zeiss Observer Z.1). Motility tracks for ≥35 cells per condition were analyzed using ImageJ software for each experiment.

### Immunofluorescence microscopy

BMDMs were seeded on gelatin (1%)-coated glass coverslips, challenged freshly egressed *T. gondii* tachyzoites (PRU A7; MOI 2) for 18 h or left unchallenged for 6 h (p-RSK) and fixed with 2% PFA. Cells were then permeabilized with 0.1% Triton X-100 in PBS and stained with phalloidin Alexa Fluor 594 (Thermo Scientific) or primary anti-p-RSK (Ser380/386) and anti-rabbit IgG Alexa Fluor 594 (Thermo Scientific) secondary antibodies and DAPI. Images were acquired on a Leica DMi8 with 63× objective.

### Adoptive transfers

Adoptive transfers were performed and analyzed as previously described (21). Briefly, BMDMs were stained with CellTracker CMTMR (2 µM) or Deep Red (1 µM) dyes (2.5 × 10^6^ cells each), washed, and challenged with indicated freshly egressed *T. gondii* GFP-expressing or CFSE-stained type II tachyzoites (PRU; MOI 1.5) in complete medium for 4 h. Cells were then washed and injected i.p. into C57BL/6 mice. Mice were sacrificed 18 h post-injection to collect spleens, mesenteric lymph nodes, and omenta. The organs were triturated, filtered through a 40 µm cell strainer, and fixed (4% PFA). Cells from the spleen were blocked with anti-CD16/32 antibody in FACS buffer (PBS/0.5% FBS/1 mM EDTA) and stained with CD11c antibody. Samples were then analyzed by flow cytometry on a BD LSRFortessa flow cytometer (BD Biosciences) and with FlowJo X (FlowJo LLC).

### Handling of publicly available data sets

ChIP- and ATAC-seq data available from NCBI GEO series GSE100738 (ATAC-seq), GSE57563 (H3K4me1), and GSE64767 (H3K4me3), which are described elsewhere, were visualized in the USCS genome browser (56, 80). RNA-seq data are taken from the ImmGen Ultra-low-input RNA-seq data set, NCBI GEO superseries GSE127267.

### Statistical analyses

All experiments were replicated to allow for statistical comparisons as stated for each experiment in the figure legends. The number of *n* denotes the number of biological replicates (adoptive transfer experiments: individual mice) or independent experiments (all other experiments). Statistical analyses were performed with R, RStudio, and packages afex (repeated-measures ANOVA), nlme [weighted least squares (WLS)], emmeans (Dunnett’s post-hoc), DAAG, and rcompanion (permutation tests). Hypothesis tests and inferential statistics used are indicated in the figure legends and were chosen based on experimental design, the hypothesis to be tested, data distribution, and statistics were to be presented. In cases of heteroscedascity due to normalization, multiple comparisons were done after WLS regression, and otherwise, ANOVA was run. Dose-dependent inhibition was tested with Spearman rank correlation. Chemotaxis analyses were done with linear regression for visualization of regression lines and, due to non-normal distribution, with one-sample permutation tests for hypothesis testing. Reported *P*-values from multiple comparisons were corrected with the Holm-Bonferroni method. Statistical significance is defined as *P* < 0.05.

## SUPPLEMENTAL MATERIAL

Fig. S1 mbio.02140-24-s0001.pdf Phenotypical and transcriptional responses of BMDMs to *T. gondii* challenge.

Fig. S2 mbio.02140-24-s0002.pdf Roles of MAP kinases, AP-1 and PU.1 in the transcriptional activation of *T. gondii*-challenged macrophages.

Fig. S3 mbio.02140-24-s0003.pdf Transcriptional impacts of AP-1 and PU.1 inhibition on BMDMs.

Fig. S4 mbio.02140-24-s0004.pdf Responses of Myd88^−/−^ Ticam^−/−^ Mavs^−/−^ macrophages to LPS.

Fig. S5 mbio.02140-24-s0005.pdf Transcriptional impacts of TEEGR, GRA16, and GRA18 mutants on macrophage activation.

Fig. S6 mbio.02140-24-s0006.pdf Gene expression and chromatin state in DCs and macrophages.

Fig. S7 mbio.02140-24-s0007.pdf Chromatin accessibility and gene expression in DCs and macrophages.

Fig. S8 mbio.02140-24-s0008.pdf Characterizations of human monocytes and monocyte-derived macrophages.

Legends mbio.02140-24-s0009.docx Supplemental figure legends.

Supplemental Tables mbio.02140-24-s0010.pdf Tables S1 to S3.

ASM does not own the copyrights to Supplemental Material that may be linked to, or accessed through, an article. The authors have granted ASM a non-exclusive, world-wide license to publish the Supplemental Material files. Please contact the corresponding author directly for reuse.
