# Supplementary figures and images for: Hypermigration of macrophages through the concerted action of GRA effectors on NF-κB/p38 signaling and host chromatin accessibility potentiates Toxoplasma dissemination
Source: mBio. 2024 Aug 29;15(10):e02140-24. doi: 10.1128/mbio.02140-24 (PMC11481493; doi:10.1128/mbio.02140-24)

Figure S1

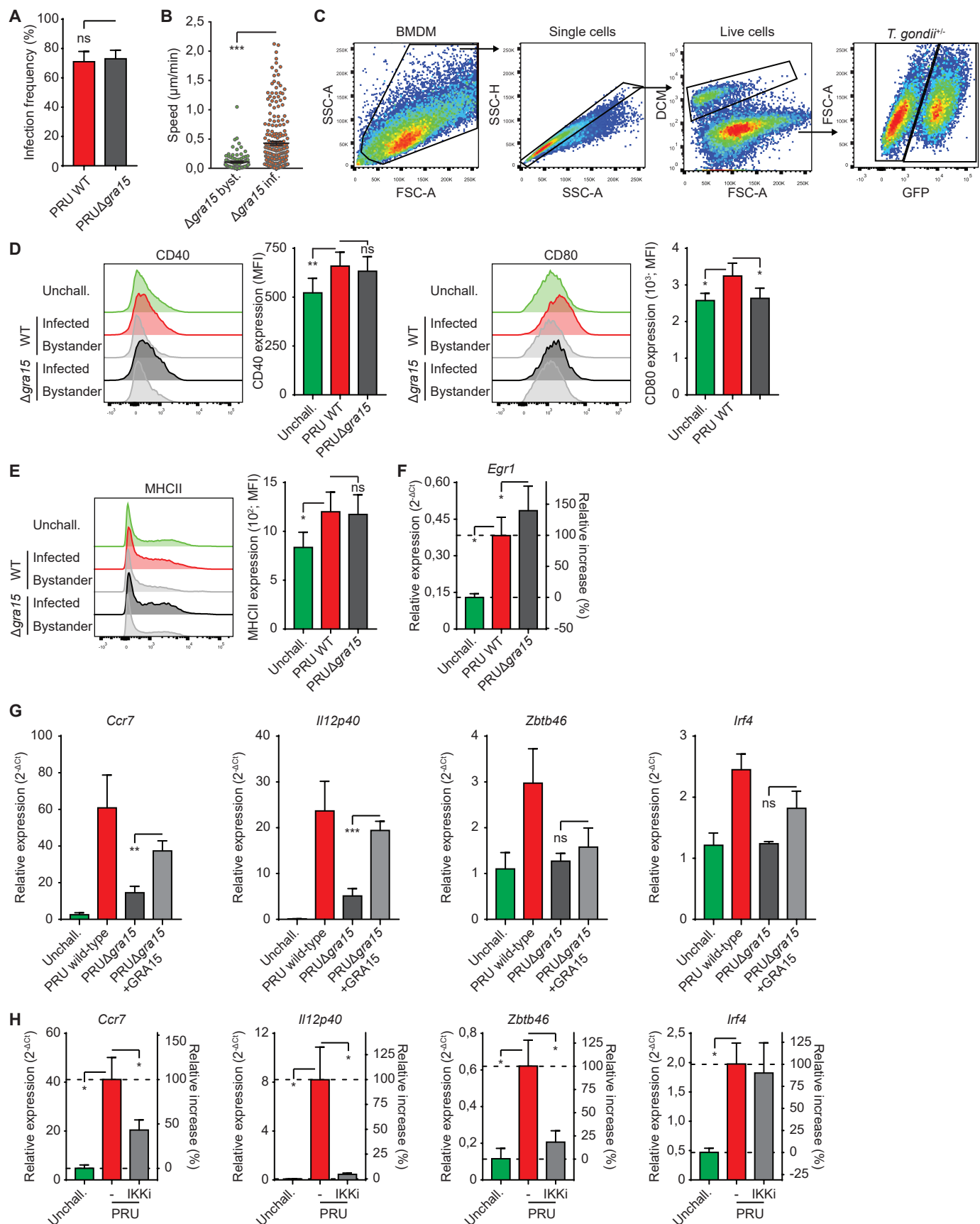

Supplement: Fig. S1 — Phenotypical and transcriptional responses of BMDMs to T. gondii challenge. [file mbio.02140-24-s0001.pdf]

Fig S2

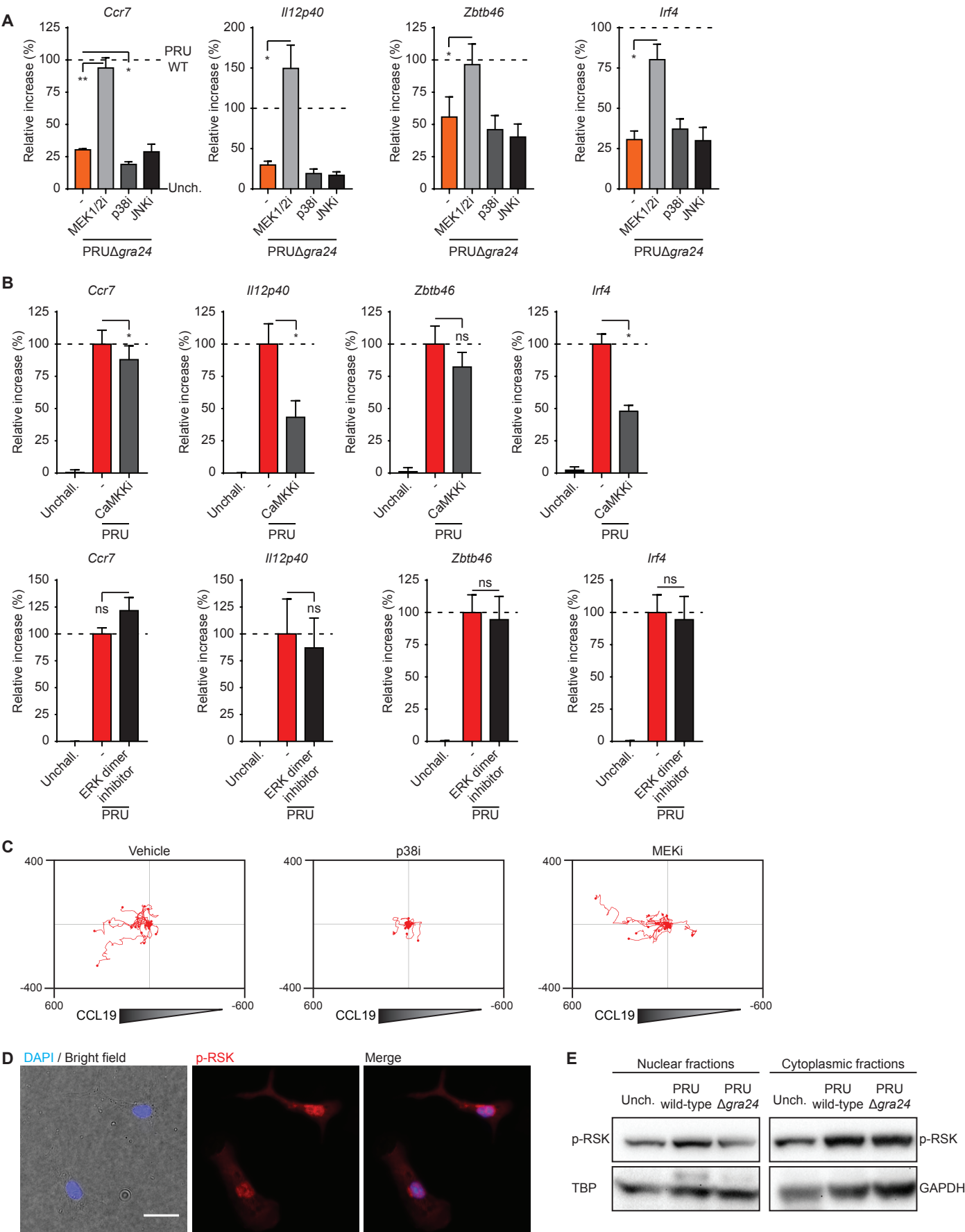

Supplement: Fig. S2 — Roles of MAP kinases, AP-1 and PU.1 in the transcriptional activation of T. gondii-challenged macrophages. [file mbio.02140-24-s0002.pdf]

Fig S3

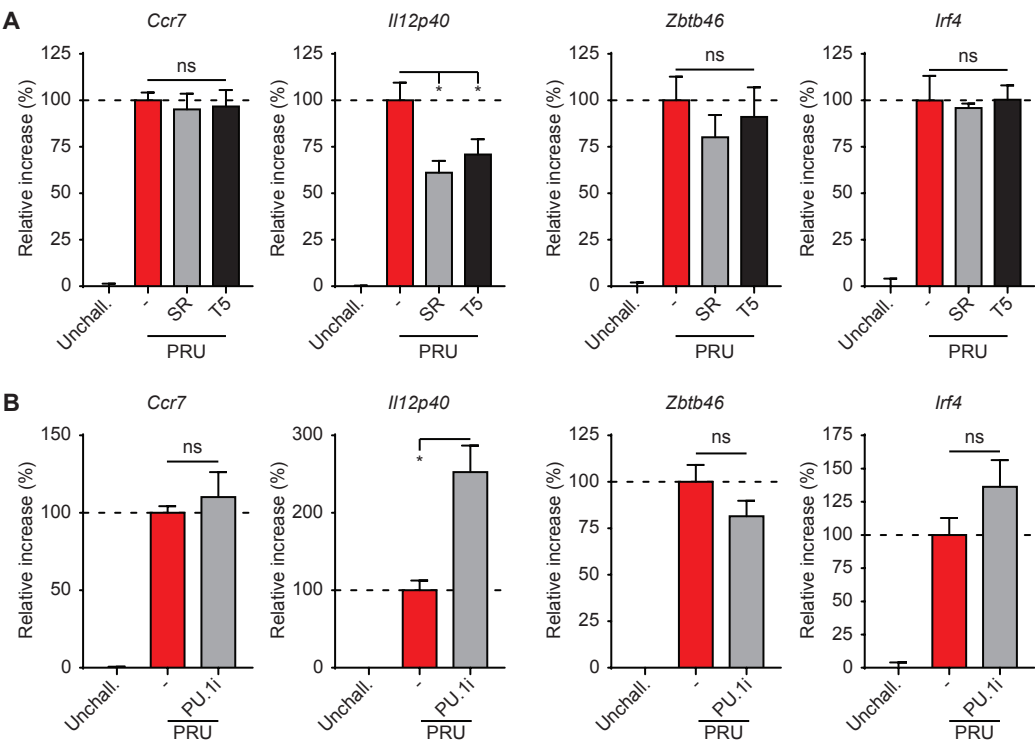

Supplement: Fig. S3 — Transcriptional impacts of AP-1 and PU.1 inhibition on BMDMs. [file mbio.02140-24-s0003.pdf]

Fig S4

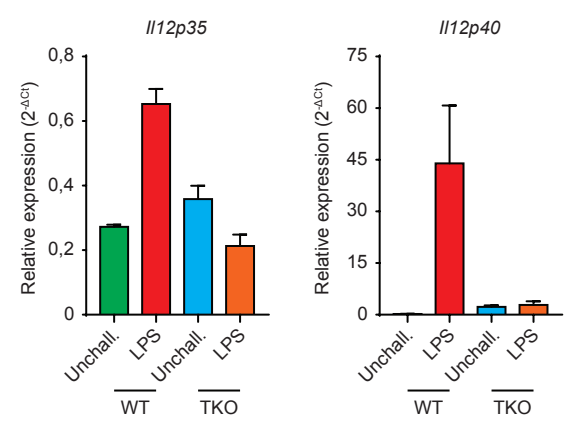

Supplement: Fig. S4 — Responses of Myd88−/− Ticam−/− Mavs−/− macrophages to LPS. [file mbio.02140-24-s0004.pdf]

Figure S5

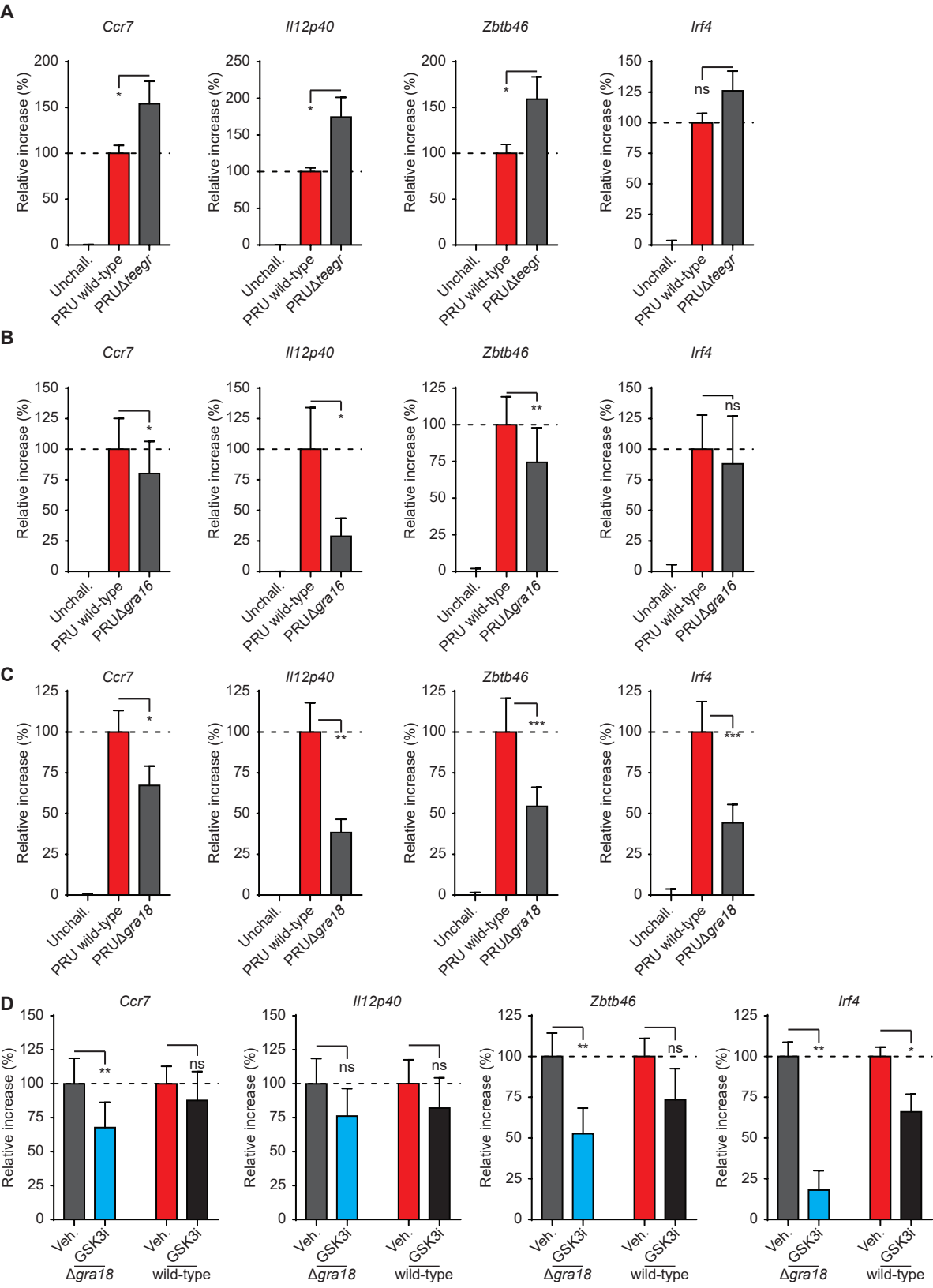

Supplement: Fig. S5 — Transcriptional impacts of TEEGR, GRA16, and GRA18 mutants on macrophage activation. [file mbio.02140-24-s0005.pdf]

Figure S6

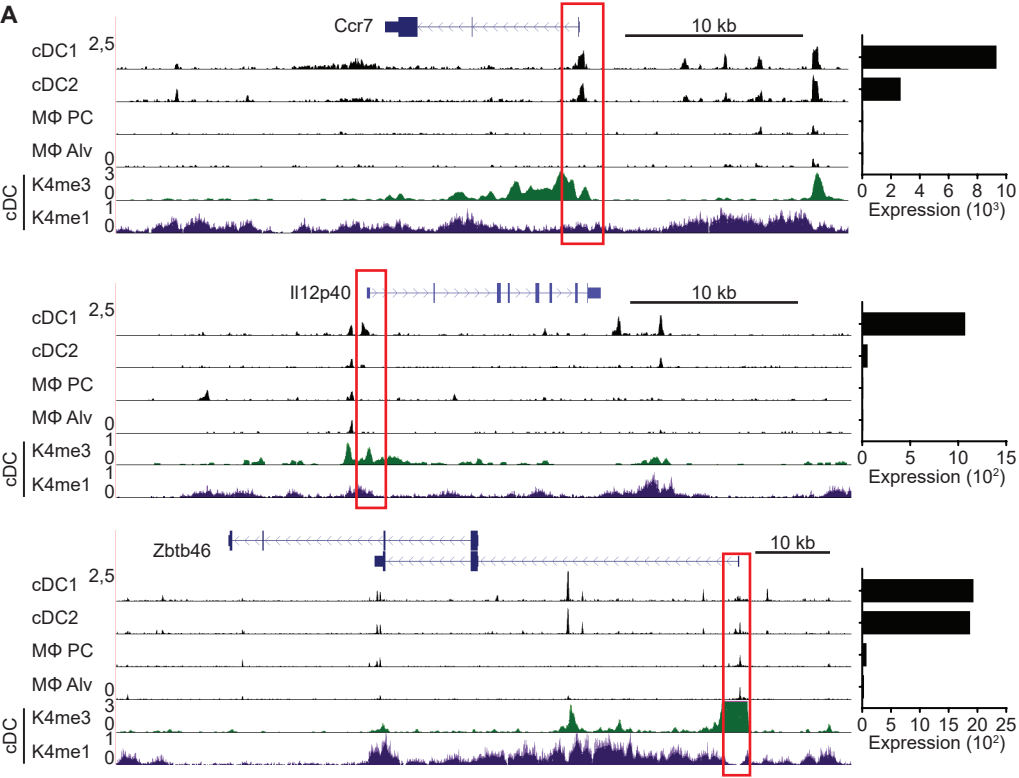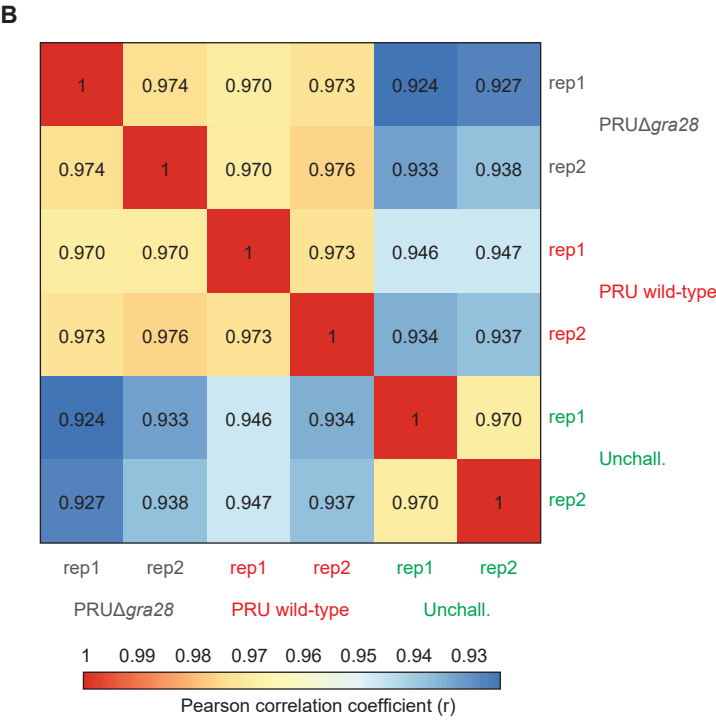

Supplement: Fig. S6 — Gene expression and chromatin state in DCs and macrophages. [file mbio.02140-24-s0006.pdf]

Figure S7

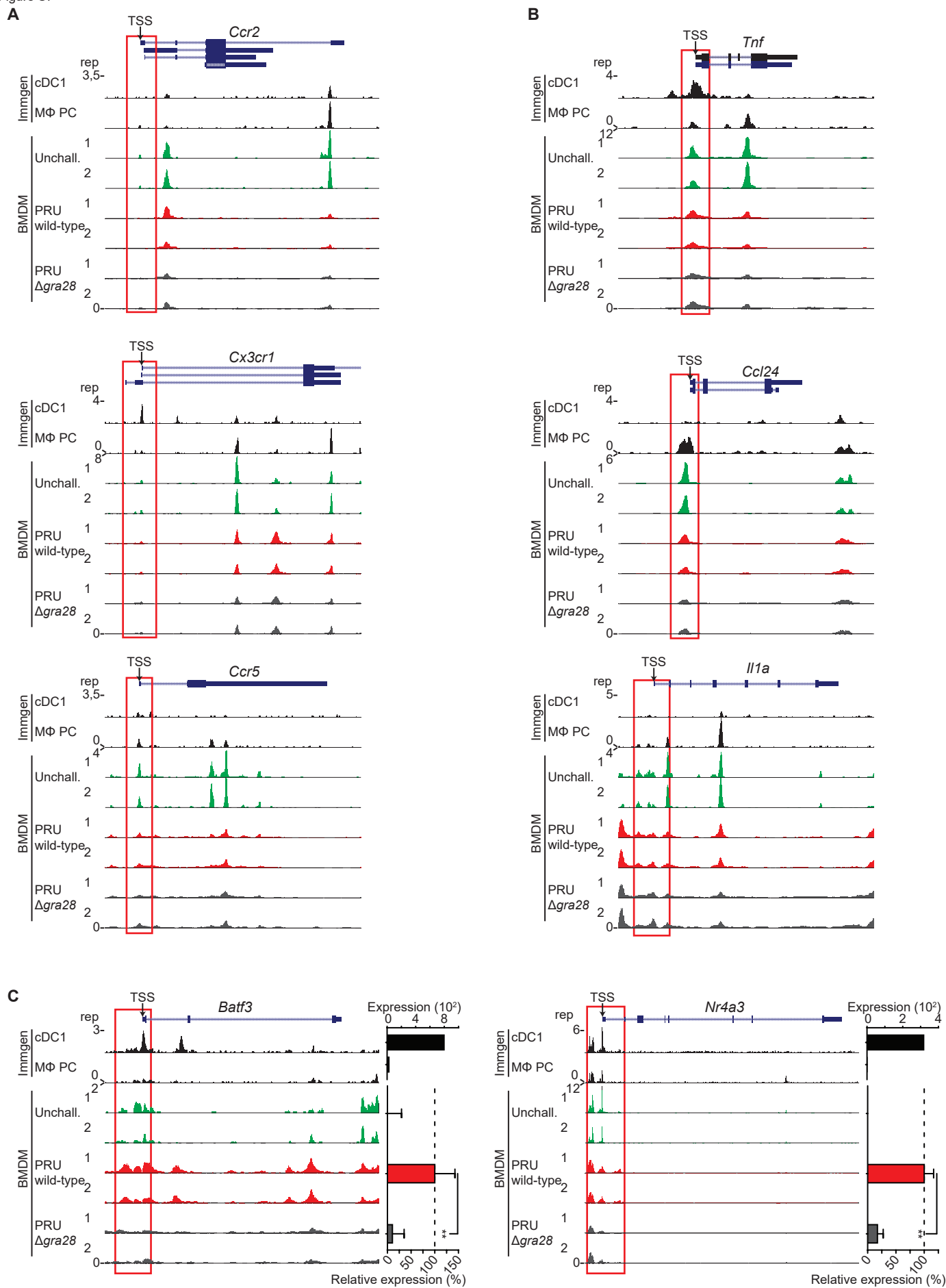

Supplement: Fig. S7 — Chromatin accessibility and gene expression in DCs and macrophages. [file mbio.02140-24-s0007.pdf]

Figure S8

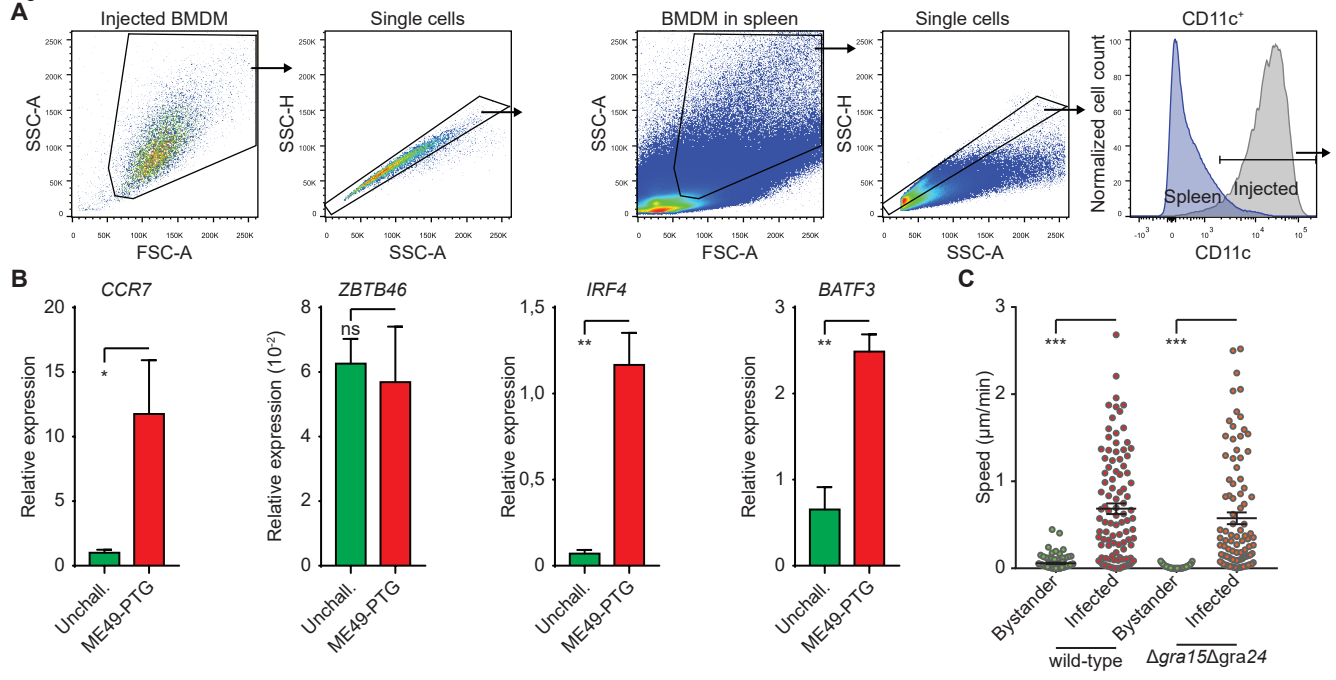

Supplement: Fig. S8 — Characterizations of human monocytes and monocyte-derived macrophages. [file mbio.02140-24-s0008.pdf]
