# Supplementary material for: Hypermigration of macrophages through the concerted action of GRA effectors on NF-κB/p38 signaling and host chromatin accessibility potentiates Toxoplasma dissemination
Source: mBio. 2024 Aug 29;15(10):e02140-24. doi: 10.1128/mbio.02140-24 (PMC11481493; doi:10.1128/mbio.02140-24)
Supplement: Legends — Supplemental figure legends. [file mbio.02140-24-s0009.docx]

**Supplementary figure legends**

**Figure S1. Phenotypical and transcriptional responses of BMDMs to *T. gondii*-challenge**

(**A**) Frequency of infected (GFP^+^) cells among BMDMs challenged with freshly egressed GFP-expressing *T. gondii* type II wild-type (WT) or GRA15-deficient (Δ*gra15*) tachyzoites for 18 h (PRU, MOI 2; n=3).

(**B**) Motility of BMDMs challenged with freshly egressed *T. gondii* type II GRA15-deficient (PRUΔ*gra15*) tachyzoites over 14-16 h in a collagen matrix with a CCL19 gradient as detailed in Methods (scale indicates µm). Dots indicate mean speed of individual cells and lines and error bars are mean ± SEM.

(**C**) Gating strategy for flow cytometric analysis of infected (GFP^+^) and bystander (GFP^-^) BMDMs challenged with freshly egressed GFP-expressing *T. gondii* type II tachyzoites.

(**D**) and (**E**) Flow cytometric analysis of anti-CD40 and CD80 (D) or MHCII (E) staining on BMDMs challenged for 18 h with freshly egressed GFP-expressing *T. gondii* type II wild-type (WT) and GRA15-deficient (Δ*gra15*) tachyzoites (PRU; MOI 1) or left unchallenged. Infected (GFP^+^) and bystander cells (GFP^-^) were analyzed. Bar graph displays the MFI of infected and unchallenged conditions (mean + SEM; n=5).

(**F**) qPCR analysis of *Egr1* cDNA from BMDMs challenged for 18 h with freshly egressed *T. gondii* type II wild-type and GRA15-deficient (Δ*gra15*) tachyzoites (PRU; MOI 2) or left unchallenged (unchall.). Displayed are relative expression (2^-ΔCt^) and the increase in expression relative to unchallenged (0%) and wild-type (100%) conditions (mean + SEM; n=5).

(**G**) qPCR analysis of *Ccr7, Il12p40, Zbtb46 and Irf4* cDNA from BMDMs derived with L929 conditioned medium and challenged for 18 h with freshly egressed *T. gondii* type II wild-type and GRA15-deficient (Δ*gra15*) tachyzoites (PRU; MOI 2) or left unchallenged (unchall.). Displayed are relative expression (2^-ΔCt^) and the increase in expression relative to unchallenged (0%) and wild-type (100%) conditions (mean + SEM; n=4).

(**H**) qPCR analysis of *Ccr7, Il12p40, Zbtb46* and *Irf4* cDNA from BMDMs challenged for 6 h with freshly egressed *T. gondii* type II wild-type tachyzoites (PRU; MOI 2) in the presence (IKKi) or absence of TPCA-1 (-) or left unchallenged (unchall.). Displayed are relative expression (2^-ΔCt^) and the increase in expression relative to unchallenged (0%) and wild-type (100%) conditions (mean + SEM; n=4).

Statistical comparisons were made with paired t-test (A), pairwise permutation test (B) or ANOVA and Dunnett’s post-hoc tests (D-H; * p ≤ 0,05, ** p ≤ 0,01, *** p ≤ 0,001, ns p > 0,05).

**Figure S2. Roles of MAP kinases, AP-1 and PU.1 in the transcriptional activation of *T. gondii* challenged macrophages**

(**A**) qPCR analysis of *Ccr7, Il12p40, Zbtb46* and *Irf4* cDNA from BMDMs challenged for 18 h with freshly egressed *T. gondii* type II GRA24-deficient tachyzoites (PRUΔ*gra24*; MOI 2) in the absence (-) or presence of MEK1/2, p38 MAPK or JNK inhibitors or left unchallenged (unchall.). Displayed is the increase in expression relative to unchallenged (0%) and *T. gondii* type II wild-type-challenged (100%) conditions (mean + SEM; n=4).

(**B**) qPCR analyses of *Ccr7, Il12p40, Zbtb46* and *Irf4* cDNA from BMDMs challenged for 18 h with *T. gondii* type II tachyzoites (PRU, MOI 2) in the absence (-) or presence of ERK1/2 dimerization inhibitor DEL-22379, CaMKK inhibitor STO-609 (CaMKKi) or left unchallenged (unchall.). Displayed is the increase in expression relative to unchallenged (0%) and wild-type-challenged (100%) conditions (mean + SEM; n=3 (DEL-22379) or 4 (STO-609)).

(**C**) Motility plots depict the displacement of BMDMs challenged with freshly egressed *T. gondii* type I tachyzoites (RH1-1; MOI 1) in the presence or absence (vehicle) of p38 MAPK (p38i) or MEK1/2 (MEKi) over 14 h in a collagen matrix with a CCL19 gradient as detailed in Methods (scale indicates µm). Infected cells (GFP^+^) were analyzed.

(**D**) Representative micrograph shows unchallenged BMDMs stained for p-RSK (S380/386, red) and nuclei (DAPI, blue). Scale bar = 10 µm.

(**E**) Western blot analysis of p-RSK (S380/386) expression in cytoplasm- and nucleus-enriched fractions of BMDMs challenged for 5 h with wild-type and GRA24-deficient (Δ*gra24*) *T. gondii* type II tachyzoites (PRU, MOI 3).

Statistical comparisons were made with ANOVA and Dunnett’s post-hoc tests (A) or paired t-test (B, C; * p ≤ 0,05, ** p ≤ 0,01, ns p > 0,05).

**Figure S3. Transcriptional impacts of AP-1 and PU.1 inhibition on DMDMs**

(**A**) and (**B**) qPCR analyses of *Ccr7, Il12p40, Zbtb46* and *Irf4* cDNA from BMDMs challenged for 18 h with *T. gondii* type II tachyzoites (PRU, MOI 2) in the absence (-) or presence of (A) AP-1 inhibitors SR 11302 (SR) and T-5224 (T5) or (B) PU.1 inhibitor DB2313. Displayed is the increase in expression relative to unchallenged (0%) and wild-type-challenged (100%) conditions (mean + SEM; n=3 (B) or 4(A)).

Statistical comparisons were made with ANOVA and Dunnett’s post-hoc tests (A) or paired t-test (B; * p ≤ 0,05, ns p > 0,05).

**Figure S4. Responses of Myd88^-/-^ Ticam^-/-^ Mavs^-/-^ macrophages to LPS**

qPCR analysis of *Il12p35* and *Il12p40* cDNA from wild-type (WT) or Myd88^-/-^ Ticam^-/-^ Mavs^-/-^ (TKO) BMDMs challenged for 18 h with LPS (10 ng/mL) or left unchallenged (unchall.). Displayed is relative expression (2^-ΔCt^) (mean + SEM; n=2).

**Figure S5. Transcriptional impacts of TEEGR, GRA16 and GRA18 mutants on macrophage activation**

(**A**), (**B**) and (**C**) qPCR analyses of *Ccr7*, *Il12p40*, *Zbtb46* and *Irf4* cDNA from BMDMs challenged for 18h (MOI2) with *T. gondii* type II PRU (wild-type), (A) TEEGR-deficient mutant (Δ*teegr*), (B) GRA16-deficient mutant (Δ*gra16*) or (C) GRA18-deficient mutant (Δ*gra18*). Displayed is the increase in expression relative to unchallenged (unchall., 0%) and wild-type (100%) challenged conditions (mean + SEM, n=5 (A; B) and n=7(C).

**(D)** Impact of GSK3β inhibition on the transcriptional activation of macrophages. qPCR analyses of *Ccr7*, *Il12p40*, *Zbtb46* and *Irf4* cDNA from BMDMs challenged with *T. gondii* wild-type or GRA18-deficient (Δ*gra18*) tachyzoites (PRU), with or without AR-A014418 (GSK3i), as in (A). Bar graphs display the increase in expression relative to untreated unchallenged (unchall., 0%) and GRA18-deficient or wild-type (100%) challenged conditions (mean + SEM). Statistical comparisons were made with Student’s t-tests for paired samples (n=5, * p ≤ 0,05, ** p ≤ 0,01, ns p > 0,05).

**Figure S6. Gene expression and chromatin state in DCs and macrophages**

(**A**) Genome tracks show peak signal intensity (y-axis) for open or closed chromatin regions and indicated histone marks at selected genes from publicly available ATAC-seq and ChIP-seq data. Bar graphs show the corresponding mRNA expression of these genes from publicly available RNA-seq data. ATAC-seq tracks are of splenic CD8+ and CD4+ DC (cDC1/cDC2), peritoneal (MΦ PC) and alveolar macrophages (MΦ Alv). H3K4me1 (K4me1) and H3K4me3 (K4me3), indicative of active promoters and enhancers (1), ChIP-seq tracks are of *in vitro*-derived Flt3L-DC (cDC). See Materials and Methods for sources and details.

(**B**) Heatmap reports Pearson correlation co-efficient based on the chromatin accessibility measured by ATAC-seq between biological replicates of each condition: BMDMs challenged for 18h with *T. gondii* wild-type or GRA28-deficient (Δ*gra28*) tachyzoites (PRU) or left unchallenged.

**Figure S7. Chromatin accessibility and gene expression in DCs and macrophages**

# (A) Genome tracks show peak signal intensity (y-axis) for open or closed chromatin regions of the *Ccr2*, *Ccr5* and *Cx3cr1* genes. For BMDMs, ATAC-seq signal from 2 separate biological replicates per condition. Upper tracks show peak signal from dendritic cells (cDC1) and peritoneal cavity macrophages (PC MΦ) extracted from Immgen publicly available dataset. Indicated is a region of interest (red outline) near the transcription start site (TSS).

(**B**) Visualization of open or closed chromatin regions of *Ccl24*, *Tnf* and *Il1a* genes as in (A).

(**C**) Visualization of open or closed chromatin regions of *Batf3* and *Nr4a3* genes as in (A) and mRNA expression from the publicly available Immgen RNA-seq dataset and qPCR presented in this paper. Statistical comparisons were made with ANOVA and Dunnett’s post-hoc tests (n=4-5, ** p ≤ 0,01).

**Figure S8. Characterizations of human monocytes and monocyte-derived macrophages**

(**A**) Gating strategy for flow cytometric detection in organs of intraperitoneally injected BMDMs (mesenteric lymph nodes, omentum) or CD11c^+^ BMDMs (spleen), based on gating of injected BMDMs as displayed. The histogram shows CD11c^+^ staining of cells extracted from spleen (blue) and injected BMDMs for reference (grey). The following gating steps are depicted in figure 7B.

(**B**) qPCR analyses of *Ccr7*, *Il12p40*, *Zbtb46* and *Irf4* cDNA from human monocytes challenged with *T. gondii* type II line ME49-PTG (18h, MOI 2). Displayed is relative expression (2^-ΔCt^) or the increase in expression relative to untreated unchallenged (unchall., 0%) and wild-type (100%) challenged conditions (mean + SEM, n=4).

(**C**) Motility of mo-macs challenged with PRU wild-type and Δ*gra15*Δ*gra24* tachyzoites (14h MOI 1) in a CCL19 gradient as detailed in Methods (scale indicates µm). Dots indicate mean speed of individual cells and lines and error bars are mean ± SEM. Statistical comparisons were made with pairwise permutation test (*** p ≤ 0,001).

1. **Zhou VW, Goren A, Bernstein BE.** 2011. Charting histone modifications and the functional organization of mammalian genomes. Nat Rev Genet **12:**7-18.
