# Supplementary material for: Hypermigration of macrophages through the concerted action of GRA effectors on NF-κB/p38 signaling and host chromatin accessibility potentiates Toxoplasma dissemination
Source: mBio. 2024 Aug 29;15(10):e02140-24. doi: 10.1128/mbio.02140-24 (PMC11481493; doi:10.1128/mbio.02140-24)
Supplement: Supplemental Tables — Tables S1 to S3. [file mbio.02140-24-s0010.pdf]

Table S1. Cell lines, mouse strains and parasite strains

**Cell lines**		
Human foreskin fibroblasts HFF-1	American Type Culture Collection	Cat# SCRC-1041 RRID:CVCL_3285
**Mouse strains**		
Mouse C57BL/6NCrl (wild-type)	Charles River Laboratories	Strain code 027 RRID:IMSR_CRL:027
Mouse C57BL/6 Myd88$^{-/-}$ Ticam$^{-/-}$ Mavs$^{-/-}$	(Erttmann et al., 2022)	N/A
**Parasite strains**		
T. gondii PruΔku80	(Braun et al., 2019)	N/A
T. gondii PruΔku80 Δ*gra28*	(Ten Hoeve et al., 2022)	N/A
T. gondii PruΔku80 Δ*myr1*	(Braun et al., 2019)	N/A
T. gondii PruΔku80 Δ*TEEGR*	(Braun et al., 2019)	N/A
T. gondii PruΔku80 Δ*gra16*	(Bougdour et al., 2013)	N/A
T. gondii PruΔku80 Δ*gra18*	(He et al., 2018)	N/A
T. gondii PruΔhpt GFP^{+} (PRU A7)	(Kim et al., 2007)	N/A
T. gondii PruΔhpt GFP^{+} Δ*gra15*	(Mukhopadhyay et al., 2020)	N/A
T. gondii Pru GFP^{+} Δ*gra15*+*gra15*	(Rosowski et al., 2011)	N/A
T. gondii PruΔhpt GFP^{+} Δ*gra24*	(Mukhopadhyay et al., 2020)	N/A
T. gondii PruΔhpt GFP^{+} Δ*gra15* Δ*gra24*	(Mukhopadhyay et al., 2020)	N/A
T. gondii ME49-PTG	(Hitziger et al., 2005)	N/A

Bougdour, A., E. Durandau, M.P. Brenier-Pinchart, P. Ortet, M. Barakat, S. Kieffer, A. Curt-Varesano, R.L. Curt-Bertini, O. Bastien, Y. Coute, H. Pelloux, and M.A. Hakimi. 2013. Host cell subversion by Toxoplasma GRA16, an exported dense granule protein that targets the host cell nucleus and alters gene expression. *Cell host & microbe* 13:489-500.

Braun, L., M.P. Brenier-Pinchart, P.M. Hammoudi, D. Cannella, S. Kieffer-Jaquinod, J. Vollaire, V. Josserand, B. Touquet, Y. Couté, I. Tardieux, A. Bougdour, and M.A. Hakimi. 2019. The Toxoplasma effector TEEGR promotes parasite persistence by modulating NF-κB signalling via EZH2. *Nat Microbiol* 4:1208-1220.

Erttmann, S.F., P. Swacha, K.M. Aung, B. Brindefalk, H. Jiang, A. Härtlova, B.E. Uhlin, S.N. Wai, and N.O. Gekara. 2022. The gut microbiota prime systemic antiviral immunity via the cGAS-STING-IFN-I axis. *Immunity* 55:847-861.e810.

He, H., M.P. Brenier-Pinchart, L. Braun, A. Kraut, B. Touquet, Y. Couté, I. Tardieux, M.A. Hakimi, and A. Bougdour. 2018. Characterization of a Toxoplasma effector uncovers an alternative GSK3/β-catenin-regulatory pathway of inflammation. *eLife* 7:

Hitziger, N., I. Dellacasa, B. Albiger, and A. Barragan. 2005. Dissemination of Toxoplasma gondii to immunoprivileged organs and role of Toll/interleukin-1 receptor signalling for host resistance assessed by in vivo bioluminescence imaging. *Cellular microbiology* 7:837-848.

Kim, S.K., A. Karasov, and J.C. Boothroyd. 2007. Bradyzoite-specific surface antigen SRS9 plays a role in maintaining Toxoplasma gondii persistence in the brain and in host control of parasite replication in the intestine. *Infection and immunity* 75:1626-1634.

Mukhopadhyay, D., D. Arranz-Solís, and J.P.J. Saeij. 2020. Toxoplasma GRA15 and GRA24 are important activators of the host innate immune response in the absence of TLR11. *PLoS pathogens* 16:e1008586.

Rosowski, E.E., D. Lu, L. Julien, L. Rodda, R.A. Gaiser, K.D. Jensen, and J.P. Saeij. 2011. Strain-specific activation of the NF-kappaB pathway by GRA15, a novel Toxoplasma gondii dense granule protein. *The Journal of experimental medicine* 208:195-212.

Ten Hoeve, A.L., L. Braun, M.E. Rodriguez, G.C. Olivera, A. Bougdour, L. Belmudes, Y. Couté, J.P.J. Saeij, M.A. Hakimi, and A. Barragan. 2022. The Toxoplasma effector GRA28 promotes parasite dissemination by inducing dendritic cell-like migratory properties in infected macrophages. *Cell host & microbe* 30:1570-1588.e1577.

Table S2. Antibodies, chemicals and kits

**Antibodies**		
PE-Cyanine7 anti-mouse CD11c (clone N418)	eBioscience	Cat# 25-0114-82 RRID:AB_469590
anti-mouse CD16/CD32 (clone 93)	eBioscience	Cat# 14-0161-82 RRID:AB_467133
Super Bright 780 anti-mouse CD40 (clone 1C10)	eBioscience	Cat# 78-0401-82 RRID:AB_2762674
Super Bright 645 anti-mouse CD80 (clone 16-10A1)	eBioscience	Cat# 64-0801-80 RRID:AB_2663120
Super Bright 600 anti-mouse CD86 (clone GL1)	eBioscience	Cat# 63-0862-80 RRID:AB_2662861
Super Bright 702 anti-mouse MHCII I-A/I-E (clone M5/114)	eBioscience	Cat# 67-5321-82 RRID:AB_2717173
Anti-TATA binding protein	Abcam	Cat# ab51841 RRID:AB_945758
Goat anti-Mouse IgG (H+L) Secondary Antibody, HRP	Thermo Fisher	Cat# 32430 RRID:AB_1185566
Anti-rabbit IgG, HRP-linked Antibody	Cell Signaling Technology	Cat# 7074 RRID:AB_2099233
Anti-phospho-IκBα (Ser32/36) (5A5)	Cell Signaling Technology	Cat# 9246 RRID:AB_2267145
Rabbit anti-p38 MAPK, phospho (Thr180 / Tyr182)	Cell Signaling Technology	Cat# 4511 RRID:AB_2139682
Anti-phospho-p90RSK (Ser380) Antibody	Cell Signaling Technology	Cat# 9341 RRID:AB_330753
**Inhibitors**		
TPCA-1, IKK-2 and STAT3 inhibitor	MedChemExpress	Cat# HY-10074
Trametinib, MEK1/2 inhibitor	Selleckchem	Cat# S2673
BIRB 796, pan-p38 MAPK inhibitor	Calbiochem	Cat# 506172
JNK-IN-8, JNK inhibitor	MedChemExpress	Cat# HY-13319
BRD7389, RSK1-3 inhibitor	MedChemExpress	Cat# HY-12185
MK2-IN-1, MK2 inhibitor	MedChemExpress	Cat# HY-12834
SR 11302, AP-1 inhibitor	MedChemExpress	Cat# HY-15870
T-5224, c-Fos/AP-1 inhibitor	MedChemExpress	Cat# HY-12270
JSH-23, NF-κB inhibitor	MedChemExpress	Cat# HY-13982
DB2313, PU.1 inhibitor	MedChemExpress	Cat# HY-124629
BIX02189, MEK5 inhibitor	AdooQ Bioscience	Cat# A10151
**Chemicals and kits**		
CellTracker Orange CMTMR Dye	Thermo Fisher	Cat# C2927
CellTracker Deep Red Dye	Thermo Fisher	Cat# C34565

CellTrace CFSE Cell Proliferation Kit	Thermo Fisher	Cat# C34554
LIVE/DEAD™ Fixable Far Red Dead Cell Stain Kit	Thermo Fisher	Cat# L34973
CD14 MicroBeads, human, 2 mL	Miltenyi Biotec	Cat # 130-050-201
MiniMACS Separation columns, type MS	Miltenyi Biotec	Cat # 130-042-201
Lymphoprep	Axis Shield Poc As	Cat# 1115754
Direct-zol RNA Miniprep kit	Zymo Research	Cat# R2052
Total RNA purification kit	Jena Bioscience	Cat# PP-210L
Recombinant Murine GM-CSF	Peprotech	Cat# 315-03
Recombinant Human GM-CSF	Peprotech	Cat# 300-03
Recombinant Murine MIP-3β (CCL19)	Peprotech	Cat# 250-27B
Recombinant Human MIP-3β (CCL19)	Peprotech	Cat# 300-29B
ATAC-Seq Kit	Active Motif	Cat# 53150

Table S3. Oligonucleotides

Primer murine Zbtb46 forward: AGAGAGCACATGAAGCGACA	(Satpathy et al., 2012)
Primer murine Zbtb46 reverse: CTGGCTGCAGACATGAACAC	(Satpathy et al., 2012)
Primer murine Irf4 forward: CTCATCACAGCTCATGTGG	(Ten Hoeve et al., 2022)
Primer murine Irf4 reverse: CCTCAGGAAATGTCCAGTG	(Ten Hoeve et al., 2022)
Primer murine Batf3 forward: AGGTCAAATCTCAGAGCCC	(Ten Hoeve et al., 2022)
Primer murine Batf3 reverse: TTCTGGGTCTGCTTCTTCC	(Ten Hoeve et al., 2022)
Primer murine Ccr7 forward: CCAGGTGTGCTTCTGCCAAG	(Ten Hoeve et al., 2022)
Primer murine Ccr7 reverse: AAAGTTCCGCACATCCTTCT	(Ten Hoeve et al., 2022)
Primer murine Nr4a3 forward: ATGGTTAAGGAAGTTGTGCG	(Ten Hoeve et al., 2022)
Primer murine Nr4a3 reverse: TTGTAGTGGGCTCTTTGGT	(Ten Hoeve et al., 2022)
Primer murine Il12p40 forward: TCCCTCAAGTTCTTTGTTCG	(ten Hoeve et al., 2019)
Primer murine Il12p40 reverse: CGCACCTTTCTGGTTACAC	(ten Hoeve et al., 2019)
Primer murine Egr1 forward: TTCAATCCTCAAGGGGAGCC	(ten Hoeve et al., 2019)
Primer murine Egr1 reverse: AAAGGACTCTGTGGTCAGGTG	(ten Hoeve et al., 2019)
Primer murine Tbp forward: GGGGAGCTGTGATGTGAAGT	(Bhandage et al., 2020)
Primer murine Tbp reverse: CCAGGAAATAATTCTGGCTCA	(Bhandage et al., 2020)
Primer murine Ipo8 forward: CTATGCTCTCGTTCAGTATGC	(Bhandage et al., 2020)

Primer murine Ipo8 reverse: GTCCGAAAGATCTCCATCCA	(Bhandage et al., 2020)
Primer human Zbtb46 forward: CGGGAAGAAGTTCACGCGG	(Wang et al., 2019)
Primer human Zbtb46 reverse: CTGCACACCTTGCACACATAC	(Wang et al., 2019)
Primer human Batf3 forward: TCCATGAGGAATATGAGAGCC	(Ten Hoeve et al., 2022)
Primer human Batf3 reverse: CTCCTTCAGTGCCTCTGTC	(Ten Hoeve et al., 2022)
Primer human Irf4 forward: CAGGATTGTTCCTGAGGGAG	(Ten Hoeve et al., 2022)
Primer human Irf4 reverse: TAGTTGTGAACCTGCTGGG	(Ten Hoeve et al., 2022)
Primer human Ccr7 forward: TCAAGACCATGACCGATACC	(Ten Hoeve et al., 2022)
Primer human Ccr7 reverse: AGGAGGAAGAGGATGTCTG	(Ten Hoeve et al., 2022)
Primer human Ipo8 forward: GCAAAGGAAGGGGAATTGAT	(ten Hoeve et al., 2019)
Primer human Ipo8 reverse: CGAAGCTCACTAGTTTTGACCC	(ten Hoeve et al., 2019)
Primer human Tbp forward: GAGCTGTGATGTGAAGTTTCC	(ten Hoeve et al., 2019)
Primer human Tbp reverse: TCTGGGTTTGATCATTCTGTAG	(ten Hoeve et al., 2019)

Bhandage, A.K., G.C. Olivera, S. Kanatani, E. Thompson, K. Loré, M. Varas-Godoy, and A. Barragan. 2020. A motogenic GABAergic system of mononuclear phagocytes facilitates dissemination of coccidian parasites. *eLife* 9:

Satpathy, A.T., W. Kc, J.C. Albring, B.T. Edelson, N.M. Kretzer, D. Bhattacharya, T.L. Murphy, and K.M. Murphy. 2012. Zbtb46 expression distinguishes classical dendritic cells and their committed progenitors from other immune lineages. *The Journal of experimental medicine* 209:1135-1152.

Ten Hoeve, A.L., L. Braun, M.E. Rodriguez, G.C. Olivera, A. Bougdour, L. Belmudes, Y. Couté, J.P.J. Saeij, M.A. Hakimi, and A. Barragan. 2022. The Toxoplasma effector GRA28 promotes parasite dissemination by inducing dendritic cell-like migratory properties in infected macrophages. *Cell host & microbe* 30:1570-1588.e1577.

ten Hoeve, A.L., M.-A. Hakimi, and A. Barragan. 2019. Sustained Egr-1 Response via p38 MAP Kinase Signaling Modulates Early Immune Responses of Dendritic Cells Parasitized by Toxoplasma gondii. *Frontiers in Cellular and Infection Microbiology* 9:

Wang, Y., H.Y. Sun, S. Kumar, M.D.M. Puerta, H. Jo, and A. Rezvan. 2019. ZBTB46 is a shear-sensitive transcription factor inhibiting endothelial cell proliferation via gene expression regulation of cell cycle proteins. *Lab Invest* 99:305-318.
